# Monoamine Oxidase Inhibitors in Toxic Models of Parkinsonism

**DOI:** 10.3390/ijms26031248

**Published:** 2025-01-31

**Authors:** Olga Buneeva, Alexei Medvedev

**Affiliations:** Institute of Biomedical Chemistry, 10 Pogodinskaya Street, 119121 Moscow, Russia; olbuneeva@gmail.com

**Keywords:** Parkinson’s disease, toxin-based models, monoamine oxidase, monoamine oxidase inhibitors

## Abstract

Monoamine oxidase inhibitors are widely used for the symptomatic treatment of Parkinson’s disease (PD). They demonstrate antiparkinsonian activity in different toxin-based models induced by 6-hydroxydopamine, 1-methyl-4-phenyl-1,2,3,6-tetrahydropyridine (MPTP), and pesticides (rotenone and paraquat). In some models, such as MPTP-induced PD, MAO inhibitors prevent the formation of the neurotoxin MPP^+^ from the protoxin MPTP. Regardless of the toxin’s nature, potent MAO inhibitors prevent dopamine loss reduction, the formation of hydrogen peroxide, hydrogen peroxide signaling, and the accumulation of hydrogen peroxide-derived reactive oxygen species responsible for the development of oxidative stress. It becomes increasingly clear that some metabolites of MAO inhibitors (e.g., the rasagiline metabolite 1-R-aminoindan) possess their own bio-pharmacological activities unrelated to the parent compound. In addition, various MAO inhibitors exhibit multitarget action, in which MAO-independent effects prevail. This opens new prospects in the development of novel therapeutics based on simultaneous actions on several prospective targets for the therapy of PD.

## 1. Introduction

Parkinson’s disease (PD) is one of the most common and the most rapidly growing neurodegenerative disorders, and it affects millions of people worldwide [1,2,3,4,5]. It is characterized by degeneration of the nigrostriatal dopaminergic system and the formation of intracellular inclusions rich in alpha-synuclein (Lewy bodies) and Lewy neurites [6,7,8,9,10,11]. In addition to such motor disorders as bradykinesia, rigidity, and tremor, PD patients show non-motor symptoms (anxiety, depression, cognitive loss, and sleep disturbance) [12,13].

To date about 100 distinct genes or loci associated with this heterogeneous disease are known [14]. Various models of PD are successfully used to study the effect of the impaired synthesis of the proteins encoded by these genes on the synaptic dysfunction, the immune deficiency, and the abnormal functioning of mitochondria, proteasomes, and lysosomes. These include transgenic animal models and viral vector models with the predominant use of mice or *Drosophila melanogaster* [15,16,17,18,19] or cell cultures [20,21,22,23]. Nevertheless, sporadic (or idiopathic) PD without any family history or an apparent genetic risk occurs much more frequently than the familial cases of the disease. Certain types of toxins cause symptoms that are similar to the symptoms of PD; this makes it possible to investigate toxin models based on different objects from cell lines of nonhuman primates [24,25,26,27] to get closer to the creation of new effective pharmacological strategies. These toxins can be subdivided into three groups: neurotoxins (6-hydroxydopamine; 6-OHDA, 1-methyl-4-phenyl-1,2,3,6-tetrahydropyridine; MPTP), pesticides (rotenone, paraquat), and endotoxins (lipopolysaccharide; LPS) [26]. The advantages and shortcomings of each model, as well as their particular pathogenic mechanisms, have been considered in several recent reviews [26,28,29,30].

**Figure 1:**
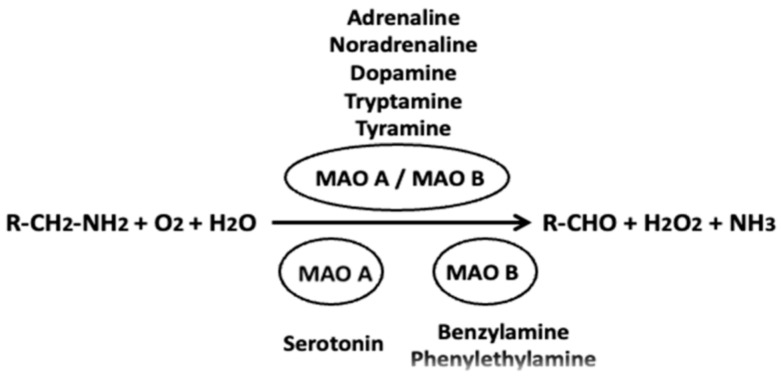
Oxidative deamination of biogenic monoamines by MAOs. Common monoamine substrates for both MAOs (MAOA/MAOB) are shown above the arrow, while preferential substrates for MAO A and MAO B are shown under the arrow. R is a radical of corresponding amine.

Monoamine oxidase (MAO, EC 1.4.3.4) is the outer mitochondrial membrane flavoenzyme that catalyzes the reaction of the oxidative deamination of mediator monoamines with the formation of the corresponding aldehydes, ammonia, and hydrogen peroxide (Figure 1). In the presence of iron, the latter can be converted into a hydroxyl radical in the Fenton reaction, thus causing cytotoxicity under pathological conditions (e.g., [30]). This MAO-dependent regulation of monoamine neurotransmitter and hydrogen peroxide levels in the brain explains the considerable interest in this enzyme as a promising drug target for the treatment of neurological disorders [31,32]. MAO exists in two isoforms (MAO A and MAO B) encoded by different genes on chromosome X [33,34]. MAO A and B proteins share 70% sequence identity [35,36], but differ in their localization, substrate specificity, and sensitivity to inhibitors. In human brain, MAO A is localized predominantly in the *locus coeruleus* and MAO B in the dorsal raphe nucleus and astrocytes, and both MAO A and B are localized in the distinct neuronal population in the hypothalamus [37]. MAO A is characterized by the greater affinity for hydroxylated amines (epinephrine (adrenaline), norepinephrine, and serotonin (5-hydroxytryptamine, 5-HT)), while MAO B predominantly oxidizes non-hydroxylated amines (beta-phenylethylamine (PEA), benzylamine) [38]. Dopamine (DA) and tyramine show similar activity for both forms of MAO. Clorgyline is known as a selective irreversible inhibitor of MAO A, whereas the irreversible inhibitors pargyline, selegiline (L-deprenyl), and rasagiline selectively inactivate MAO B [32,38,39,40].

Initially, the therapeutic effect of MAO inhibitors in the therapy of PD was considered in the context of DA loss reduction [32]. However, the neuroprotective action of MAO inhibitors goes beyond reducing the loss of striatal DA. In particular, MAO B inhibitors protect neurons by the induction of antiapoptotic and neurotrophic factors, preventing mitochondrial permeability transition and nuclear DNA fragmentation [41]. MAO B inhibition facilitates the secretion of detergent-insoluble alpha-synuclein, preventing its intracellular accumulation and the formation of aggregates—the characteristic pathological hallmark of PD [42].

Various MAO inhibitors are already used as antiparkinsonian drugs [38,43,44,45]; however, the review of MAO inhibitors in toxic models of PD could help to shed light on their efficacy and molecular mechanisms of action.

## 2. MAO Inhibitors in 6-OHDA-Based Models

5-(2-aminoethyl)-1,2,4-benzenetriol) (6-hydroxydophamine; 6-OHDA) is a neurotoxin which has long shown its ability to induce neurodegeneration of the nigrostriatal system [46,47]. Since it cannot cross the blood–brain barrier, it is administered directly into certain regions of the brain in 6-OHDA-based animal models of PD. Numerous studies have shown that 6-OHDA in low concentrations acts specifically on the dopaminergic neurons through its DA transporter-mediated uptake, and 6-OHDA in high concentrations does not need this specific transporter [48,49,50]. Although the toxic model of PD based on 6-OHDA does not reproduce all signs and symptoms of PD (e.g., it lacks Lewy bodies), it nevertheless includes many cellular processes altered in PD: oxidative stress, neuroinflammation, DNA damage, and apoptosis [50,51,52,53,54]. The 6-OHDA neurotoxicity involves various mechanisms. Due to its instability at neutral pH, 6-OHDA rapidly undergoes autoxidation accompanied by the formation of different toxic and reactive oxygen species (ROS) compounds [50,53]. It causes iron release from the iron-storage protein ferritin and some other proteins [55,56], thus inducing the formation of free radicals via the Fenton reaction [50]. Moreover, 6-OHDA directly inhibits mitochondrial complexes I and IV independently of the free radical mechanism [51,57]. Inhibition of mitochondrial electron transport may result in the shortage of ATP, thus causing the disruption of calcium homeostasis, cytochrome *c* release, mitochondrial swelling, caspase activation, and, as a result, apoptosis or necrosis [50,58,59]. Indeed, in many PD models employing different organisms and cells, 6-OHDA was shown to induce apoptosis [60,61,62,63]. 6-OHDA reduces disulfide bonds in proteins [50] and influences the activity of various multifunctional enzymes, such as glyceraldehyde 3-phosphate dehydrogenase (GAPDH) [64]. The latter not only functions as a glycolytic enzyme but also takes part in DNA repair. 6-OHDA influences chromatin structure by ROS-associated histone modifications [65].

Proteomic studies of 6-OHDA-treated rats confirmed the effect of this toxin on energy metabolism, calcium homeostasis, antioxidation, and cytoskeletal function. 6-OHDA increased the expression of prohibitin, peroxiredoxin 2, and components of mitochondrial complexes I and III and reduced calreticulin and calmodulin expressions in substantia nigra (SN) or striatum [66,67].

Since 6-OHDA has been detected in body fluids and brain biopsy samples of PD patients [68,69] and in animal models [70,71], its endogenous (enzymatic or non-enzymatic) formation, including its probable synthesis by gut microbiome enzymes [50], cannot be ruled out.

As DA is a substrate of both forms of MAO, and because levodopa (L-DOPA, L-3,4 dihydroxyphenylalanine), the immediate DA precursor, is still the main medication to treat PD [72], a lot of investigations have been devoted to the effect of MAO A and MAO B inhibitors on the metabolism of intrinsic DA and DA derived from levodopa. Selective inhibitors of MAO A (clorgyline) or MAO B (selegiline (L-deprenyl) or rasagiline) were used to clarify the contribution of each form of MAO in the catabolism of DA synthesized from exogenous levodopa in the striatum of 6-OHDA-treated rats. The results of these studies suggest that the main mechanism for the catabolism of striatal DA (including the conditions of the presence of exogenous levodopa) includes its deamination by MAO A, and in the lesioned striatum, it is mostly deaminated by MAO A in medium spiny neurons and, to a small extent, by MAO B in both medium spiny neurons and glia [73,74,75].

In contrast to early reports demonstrating the potentiation of 6-OHDA-induced brain DA depletion by administration of the irreversible MAO (B) inhibitor pargyline [76], convincing evidence exists that MAO inhibitors exhibit protective effects in this PD model. The protective effects of clorgyline, L-deprenyl (selegiline), and rasagiline in 6-OHDA-based models of PD are summarized in Table 1. In most of the models, all the inhibitors ameliorated motor impairments and reduced the level of oxidative stress. Rasagiline showed an additional antioxidant property, not only that resulting from MAO inhibition; this was possibly related to its ability to enhance expression of the antioxidant enzymes superoxide dismutase and catalase [77,78].

As the metabolic product of rasagiline (1-R-aminoindan) exhibits neuroprotective characteristics [83,87,90], in contrast to the neurotoxic products of the metabolism of selegiline (L-amphetamine and L-methamphetamine) [100], rasagiline seems to be more promising as the antiparkinsonian drug.

A genomic and proteomic study of the action of these two MAO inhibitors in the rat midbrain (one of the very few genomic and proteomic studies of the MAO inhibitor effects in neurotoxic-based models of PD) revealed that selegiline and rasagiline altered the expression of genes encoding many proteins involved in various cellular pathways. These were the proteins involved in neuronal differentiation; cell survival and death; protection from oxidative stress; metabolism of proteins, carbohydrates, and lipids; cell signaling; and enzyme activity regulation. Some proteins, which play a key role in glycolysis, oxidative stress protection, and cell signaling, exhibited differential expression in the midbrains of rats treated with selegiline or rasagiline. This suggests that selegiline and rasagiline display moderately different patterns of molecular effects and that their action is not limited to MAO inhibition [89].

VAR (5-[2-(methyl-prop-2-ynyl-amino)- ethyl]-quinolin-8-ol dihydrochloride), the iron-chelating inhibitor of MAO A and B, attenuated motor impairments in rats, significantly reduced the loss of striatal DA, and elevated the levels of serotonin [99].

In the mouse model, reversible MAO A inhibitor afobazole (4-[2-[(6-ethoxy-1*H*-benzimidazol-2-yl)sulfanyl]ethyl]morpholine) [101] normalized motor dysfunction, restored the DA level in the striatum and did not affect the contents of norepinephrine, serotonin, or its metabolites [92,93].

Animal and cell 6-OHDA models showed the protective effect of beta-carbolines (harmaline (7-methoxy-1-methyl-4,9-dihydro-3H-pyrido[3,4-b] indole), harmalol (1-methyl-4,9-dihydro-3H-pyrido[3,4-b] indol-7-ol), and harmine (7-methoxy-1-methyl-9H-pyrido[3,4-b] indole)). These reversible MAO A inhibitors protected rat brain mitochondria and synaptosomes against oxidative damage, decreased the alteration of mitochondrial swelling and membrane potential, inhibited the electron flow in mitochondria, reversed the depression of synaptosomal calcium uptake, and inhibited catecholamine-induced thioredoxin reductase inhibition, thiol oxidation, and carbonyl formation in mitochondria and synaptosomes. In a model with PC12 cells, the compounds attenuated the loss of cell viability without a significant cytotoxic effect. Harmaline and harmalol reduced the catecholamine-induced loss of the transmembrane potential of the cells [91].

The animal models based on the use of 6-OHDA and safinamide ((2S)-2-[[4-[(3-fluorophenyl) methoxy] phenyl] methylamino] propanamide) revealed the specific effects of this selective reversible MAO B inhibitor and sodium channel blocker. Safinamide did not have an influence on the levodopa-induced involuntary movements but prevented the levodopa-induced increase in striatal glutamate associated with dyskinesia appearance. Safinamide therapy suppressed microglial activation and protected dopaminergic neurons in the ipsilateral SN from degeneration. Safinamide reduced the neuronal firing rate and the synaptic currents of striatal projection neurons in a dose-dependent manner [95,96,97].

## 3. MAO Inhibitors in MPTP-Based Models

In the early 1980s, it was found that the dophaminergic neurotoxin 1-methyl-4-phenyl-1,2,3,6-tetrehydropiridine (MPTP), an analog of the narcotic meperidine, caused symptoms similar to those of PD [102]. Nowadays, MPTP is broadly used in toxic models of PD [54,103,104,105,106]. Due to its lipophilicity, it easily crosses the blood–brain barrier. In the central nervous system, the MAO B of glial astrocytes oxidizes it to an intermediate metabolite, 1-methyl-4-phenyl-2,3-dihydropyridine, which is further converted to the toxic 1-methyl-4-phenylpyridinium (MPP^+^) [3,107]. This active neurotoxin is released from the astrocytes; then, it is transferred to dopaminergic neurons via the DA transporter. Moreover, the redistribution of MPP^+^ through the transporter is a key factor in MPP^+^ toxicity [108]. Several cellular mechanisms are responsible for the death of dopaminergic neurons in SN and striatum. MPP^+^ inhibits mitochondrial complex I, thus blocking ATP synthesis and increasing production of ROS, leading to mitochondrial pore opening s and cytochrome *c* leakage. This triggers the activation of specific caspases and other proapoptotic factors [109,110]. The increase in ROS production causes lipid peroxidation, DNA damage, and protein cross-linkage [111,112]. In addition, MPP^+^ triggers an inflammation pathway, when specific proinflammatory factors are activated [113], and a glutamatergic pathway, causing an increase in extracellular glutamate in the SN and striatum [106].

In addition, the high concentration of ROS causes the excessive synthesis of alpha-synuclein and its aggregation and the production of toxic alpha-synuclein oligomers, which inhibit the ubiquitin-proteasome and autophagy systems [114,115]. As a result, Lewy body-like structures are formed. MPTP selectively augments MAO B but not MAO A protein levels [116]. As was shown in a mouse model, MPTP treatment enhanced the specific interaction between endogenous alpha-synuclein and MAO B (but not MAO A) and stimulated its enzymatic activity. This triggered the activation of asparagine endopeptidase and subsequent alpha-synuclein cleavage at N103, promoting its aggregation and neurotoxicity and leading to the degeneration of DA neurons [116]. Proteomic and transcriptomic studies, conducted using MPTP-based rodent models, demonstrated the effects of this toxin on different cell pathways: glycolysis and energy generation, mitochondrial function and response to oxidative stress, neurotransmitter release, apoptosis, calcium signaling, ubiquitin-proteasome system, cytoskeletal assembly, and Lewy body formation [117,118,119,120,121,122,123]. The quantitative analysis revealed more than 100 mitochondrial proteins which displayed significant changes in relative abundance in MPTP-treated mice compared to the controls [121]. In the isolated mitochondria from neuroblastoma cells, MPTP also caused changes in the relative quantities of the proteins of different cell functions: chaperones, metabolic enzymes, oxidative phosphorylation-related proteins, an inner mitochondrial membrane protein (mitofilin), and an outer mitochondrial membrane protein (VDAC1) [124]. Using different courses of sequential MPTP administration, it was possible to model preclinical (asymptomatic) and clinical (early symptomatic) stages of PD [125,126]. However, the effect of MAO inhibitors on the manifestation of these forms of PD has not been investigated yet. Table 2 summarizes effects of MAO inhibitors in various MPTP-based toxic models of Parkinson’s disease.

In contrast to the differences in the effects of selegiline and rasagiline in 6-OHDA rat models [132], some studies revealed a similar impact of these two MAO inhibitors in the MPTP non-human primate model. In the latter case, there were no significant differences between rasagiline/MPTP- and selegiline/MPTP-treated animals with respect to the severity of motor impairment, the decrease in dopaminergic cells in the SN, and the striatal DA levels [132]. In addition to selegiline, the same effect on the prevention of the reduction in the content of DA in the striatum in the mouse MPTP model was caused by non-selective MAO inhibitors pargyline (N-benzyl-N-methylprop-2-yn-1-amine), nialamide (N-benzyl-3-[2-(pyridine-4-carbonyl) hydrazinyl] propanamide), and tranylcypromine ((1R,2S)-2- phenylcyclo- propan-1-amine) [131] (Table 2).

In mouse MPTP models, different MAO inhibitors demonstrated the increase in the level of tyrosine hydroxylase, the enzyme catalyzing the rate-limiting reaction of DA and noradrenaline biosynthesis. These are rasagiline [135]; the iron-chelating MAO A and B inhibitors VAR [99] and M30 ([5-(*N*-methyl-*N*-propargyl- amino-methyl)- 8-hydroxyquinone]) [137,138]; MAO B inhibitor lamotrigine (6-(2,3-dichloro- phenyl)-1,2,4-triazine-3,5-diamine) [141]; MAO B inhibitor MT-20R (a derivative of ladostigil, [(3R)-3-(prop-2-ynylamino)indan-5-yl]-N-propylcarbamate) [139]; and MAO B inhibitor catalpol ((2S,3R,4S,5S,6R)-2-[[(1S,2S,4S,5S,6R,10S)-5-hydroxy-2-(hydroxy methyl)-3,9-dioxatricyclo[4.4.0.02,4]dec-7-en-10-yl]oxy]-6-(hydroxymethyl)oxane-3,4,5-triol), an iridoid glycoside present in the roots of Rehmannia glutinosa, the traditional Chinese medicinal herb [150] (see Table 2).

In addition, cell MPTP models revealed that catalpol not only prevented the inhibition of mitochondrial complex I activity and the loss of mitochondrial membrane potential but also reversed the intracellular calcium level and ROS accumulation [151] and reduced the content of lipid peroxide and increased the activity of glutathione peroxidase and superoxide dismutase [152].

The protective effect of beta-carbolines (harmaline, harmalol, and harmine), which are MAO A inhibitors, on oxidative neuronal damage was demonstrated in mouse MPTP and cell MPTP models. Beta-carbolines attenuated an increase in the MPTP treatment activities of superoxide dismutase, catalase, glutathione peroxidase, and the levels of malondialdehyde (a highly reactive bifunctional molecule, which is a final product of membrane lipid peroxidation) and carbonyls in mouse brains. Harmalol reduced the MPTP effect on the enzyme activities and formation of tissue peroxidation products. Harmaline, harmalol, and harmine attenuated the MPP^+^-induced inhibition of electron flow and membrane potential formation and the DA-induced thiol oxidation and carbonyl formation in mitochondria [142].

Beta-carbolines prevented the loss of cell viability in PC12 cells treated with MPP^+^, reduced the condensation and fragmentation of nuclei, and inhibited the decrease in mitochondrial transmembrane potential, cytochrome *c* release, activation of caspase-3, ROS formation, and depletion of GSH caused by MPP^+^ [143].

The neuroprotective effect of a competitive MAO B inhibitor isatin (see Table 2) also includes neuroprotective effects that could hardly be linked to MAO B inhibition. This endogenous regulator found in tissues and biological fluids of humans and animals exhibits a broad range of biological activities mediated by isatin-responsive genes [153] and numerous isatin-binding proteins [154,155].

It should be noted that there are enough reports of genomic and proteomic studies of toxic and genetic models of PD in the literature (for a review, see [156]); nevertheless, there is limited information on the effect of MAO inhibitors in these models. The proteomic analysis revealed the effect of MPTP administration on the repertoire of brain isatin-binding proteins. Proteomic profiling of mouse brains resulted in the identification of 96 isatin-binding proteins of different functions: enzymes involved in energy generation and carbohydrate metabolism; proteins of cytoskeleton formation and exocytosis; proteins of signal transduction and regulation of enzyme activity; antioxidant and protective proteins; regulators of gene expression, cell division, and differentiation; enzymes involved in the metabolism of proteins, amino acids, and other nitrogenous compounds; and enzymes of lipid metabolism [119]. The development of MPTP-induced locomotor impairments was accompanied by a decrease in the number of isatin-binding proteins to 63. Seven days after MPTP administration, the number of isatin-binding proteins increased and reached the control level. The profiles of isatin-binding proteins were rather specific for each group of mice (the specific proteins of each group represented 60% - 80% of the total). The major changes were found in the groups of isatin-binding proteins involved in cytoskeleton formation and exocytosis, the regulation of gene expression, and cell division and differentiation and proteins involved in signal transduction [119].

Co-administration of isatin and MPTP to mice prevented MPTP-induced inactivation of MAO B and influenced the profile of brain mitochondrial proteins binding to proteasome ubiquitin receptors (Rpn10 or Rpn13 subunits of the regulatory sub-particle of proteasome) [117,147].

A single-dose administration of MPTP resulted in a decrease in the total number of mitochondrial ubiquitinated proteins and an increase in the number of oxidized mitochondrial proteins containing the ubiquitin signature (KεGG). A comparison of ubiquitinated proteins of mouse brain mitochondrial fraction and mouse brain mitochondrial proteins bound to the Rpn10 proteasome subunit did not reveal any common proteins. This suggests that the ubiquitination of brain mitochondrial proteins is not directly related to their degradation in the proteasomes. Proteomic profiling of brain isatin-binding proteins identified enzymes involved in the functioning of the ubiquitin-conjugating system. The mapping of the identified isatin-binding proteins to known metabolic pathways indicated their participation in the parkin (E3 ubiquitin ligase)-associated pathway. The functional links involving brain mitochondrial ubiquitinated proteins were found only in the group of animals with the MPTP-induced parkinsonism, but not in animals treated with MPTP/isatin or isatin only. Thus, the neuroprotective effect of isatin may be associated with the impaired functional relationships of proteins targeted to the subsequent degradation [118].

## 4. MAO Inhibitors in Rotenone- and Paraquat-Based Models

Rotenone ((2*R*,6a*S*,12a*S*)-1,2,6,6a,12,12a-hexahydro-2-isopropenyl-8,9-dimethoxy- chromeno[3,4-*b*] furo[2,3-*h*] chromen-6-one) is a naturally occurring pesticide found in the roots of plants of the Leguminosae family. It is used in cellular models of PD and in animal, non-mammalian (e.g., the nematode *Caenorhabditis elegans*, *Drosophila melanogaster fly*, zebrafish, and caracol *Lymnaea stagnalis*), and mammalian (e.g., rat and mouse) models [157,158,159,160]. Being a lipophilic molecule, rotenone easily crosses the blood–brain barrier and reproduces many motor symptoms and histopathological features of PD, including the formation of synuclein-positive cytoplasmic inclusions [161,162,163,164]. Different cellular mechanisms involved in rotenone-induced neurodegeneration are detailed in several reviews. These include the following: the blocking of the mitochondrial electron transport chain through complex I inhibition, resulting in reduced ATP production and an ROS increase; the inhibition of NO production; the dysfunction of the ubiquitin-proteasome and autophagy systems; the promotion of the release of proinflammatory cytokines, which provoke neuroinflammation; the depolarization of microtubules; lipid peroxidation; the dysfunction of DNA repair; and the inducing of apoptotic and necrotic cell death [157,158,160,165,166,167]. More than 100 proteins displayed significant differences in their relative abundance in the proteomic studies of the rotenone effect in various cell models [168,169,170,171]. Comparative proteomic identification of the brain proteins of control rats and rats with rotenone-induced parkinsonism revealed quantitative changes in 86 proteins, most of them involved in signal transduction, the regulation of enzyme activity, cytoskeleton formation, and exocytosis, energy generation, and carbohydrate metabolism [168]. Five days after the last administration of rotenone, the altered relative content was found for 120 proteins. Although most of these proteins were associated with neurodegeneration, only two of the proteins were common for both groups of rotenone-treated animals (GAPDH and subunit B of V-type proton ATPase) [172].

Paraquat (1,1′-dimethyl-4,4′-bipyridinium), one of the widely used herbicides, has a chemical structure similar to that of MPP^+^. Paraquat is taken up into the brain by the neutral amino acid transporter, then transported into striatal cells in a Na^+^-dependent manner [173]. Paraquat PD models with the use of cells and animals have shown that paraquat inhibits mitochondrial complex I activity and activates nitric oxide synthase, microglial NADH oxidase 2, causing an increase in ROS production and oxidative stress in both the cytosol and mitochondria. A model of mice treated with paraquat and another pesticide, maneb, has shown that the release of cytochrome *c* from mitochondria causes the formation of alpha-synuclein radicals (cytochrome *c* acts as peroxidase), which induces alpha-synuclein aggregation [174]. Paraquat causes alterations in the pentose phosphate pathway metabolome. Paraquat inactivates tyrosine hydroxylase, a rate-limiting enzyme in DA synthesis. It decreases the glutathione level, increases lipid peroxidation, and activates caspases, leading to apoptosis [27,157,165,175,176,177,178]. Differential protein expression patterns in a control and paraquat- and maneb-exposed mice were revealed in the following works [179,180]. Table 3 summarizes the known effects of MAO inhibitors in various pesticide-based toxic models of Parkinson’s disease.

Rotenone models have shown a role of MAO A in neurodegeneration and MAO A inhibition in neuroprotection. Indeed, in a rotenone-based cell model, MAO A knockdown in SH-SY5Y cells increased basal complex I activity and levels of anti-apoptotic factor Bcl-2 and protected against rotenone-induced ROS, glutathione depletion, and caspase-3 activation [188]. This is consistent with the neuroprotective effect of afobazole in a rotenone-induced PD model in rats. Afobazole is a moderate MAO A inhibitor lacking any MAO B inhibitory activity (at least in the range of pharmacologically relevant concentrations) [189].

Nevertheless, most of these models are devoted to the studies of MAO B inhibitors. Thus, in rat rotenone models, deprenyl (selegiline) administration caused the restoration of locomotor impairments, dose-dependently attenuated rotenone-induced reductions in complex-I activity and glutathione levels in the SN and tyrosine hydroxylase immunoreactivity in the striatum or SN, and increased the activities of the cytosolic antioxidant enzymes superoxide dismutase and catalase [181]. Deprenyl inhibited the proliferation and activation of glial cells and inhibited autophagy of nerve cells in the SN, preventing the expression of autophagy-related protein Beclin1 and microtubule-associated protein 1 light chain 3 [182,183]. The protective effect of deprenyl was also shown in a paraquat rat model. As in the rotenone models, deprenyl exhibited the restoration of locomotor activity and an increase in the striatal DA level [184].

Rasagiline, another irreversible inhibitor of MAO B, demonstrated the protective effect in in vitro paraquat models. Rasagiline prevented SHSY5Y cell death by reducing caspase 3 activation and superoxide generation, ameliorating the fall in mitochondrial membrane potential, and increasing glutathione levels [185].

The (hetero)arylalkenylpropargylamine irreversible inhibitors of MAO B exhibited a protective effect in a PC12 cell-based rotenone model and rat striatum slices. The compounds prevented pathological DA release (induced by oxidative stress) and the formation of toxic dopamine quinone and rescued tyrosine hydroxylase positive neurons in the SN [98,133].

In a rat rotenone model, the endogenous regulator isatin significantly influenced the relative content of some brain isatin-binding proteins either immediately after the end of rotenone treatment, or 5 days later. In the former case, the neuroprotective effect of isatin was most pronounced in 2-oxoglutarate dehydrogenase (E1 component of the multienzyme mitochondrial complex), whose relative content increased 11-fold after isatin treatment. Five days after the course of treatment with rotenone, changes in the relative content of 16 proteins were observed as compared to the control, and only two of them (GAPDH and subunit B of V-type proton ATPase) were isatin-binding; their relative content also changed immediately after the end of the course of treatment with rotenone. All the isatin-binding proteins with quantitative changes were associated to varying degrees with neurodegeneration (many with Parkinson’s and Alzheimer’s diseases) [187].

MAO B inhibitor isatin or MAO A inhibitor afobazole caused neuroprotective effects on locomotor impairments and to varying degrees changed the relative content of brain mitochondrial proteins (most of them were components of the cytochrome *c* oxidase complex and voltage-dependent ion channels) and some brain proteins associated with neurodegeneration (DJ-1 protein, GAPDH, TRIM2 (E3-ubiquitin ligase), the inner mitochondrial membrane receptor Prohibitin-2, wolframin, and components of voltage-dependent ion channels). The most pronounced results were obtained for synuclein, which is the hallmark of PD [186].

## 5. MAO-Independent Effects of MAO Inhibitors

As can be seen, the same MAO inhibitors act differently in different models of PD. Some effects of MAO inhibitors in cell and animal PD models are mostly associated with the decrease in the MAO activity, followed by reduction in hydrogen peroxide levels, decreased hydrogen peroxide signaling, decreased ROS generation, and DA protection. This is probably true in the case of L-deprenyl (selegiline), clorgyline, rasagiline, and some other inhibitors. However, the protective action of these compounds is not limited to MAO inhibition [41,190] because even highly selective MAO inhibitors act on more than one particular target. For example, the irreversible inhibition of both types of MAO (A+B) by a large dose of pargyline [191] had a significant impact on the binding pattern of [^3^H]-Isatin in rat brain but did not abolish it [192].

The 3-week administration of L-deprenyl to rats caused a significant increase in Cu,Zn-SOD mRNA in the nucleus accumbent (NA), striatum, and globus pallidus (GP) [193]. A similar study has shown that the 3-week administration of L-deprenyl decreased SOD mRNA and GAPDH mRNA in mouse cortex [194]. Together with data on clorgyline affinity towards sigma receptor binding sites [195], these and other reports indicate a significant role of the MAO-independent effects of MAO inhibitors.

Isatin administration influenced the expression of more than 850 genes in brain hemispheres (including 433 upregulated and 418 downregulated genes), but none of them could account for the changes in the differentially expressed proteins [153].

In many animal and cell models, selegiline and rasagiline caused antiapoptotic protective effects against various toxins: they upregulated the antiapoptotic factors Bcl-2 and Bcl-w and downregulated the proapoptotic factors Bad and Bax [196,197,198]. In MPTP SH-SY5Y cell models, selegiline, without any influence on MAO activity, augmented the gene induction of thioredoxin, leading to elevated expression of mitochondrial antioxidative manganese superoxide dismutase and antiapoptotic Bcl-2 [130]. In SH-SY5Y cells and mouse models, rasagiline not only inhibited MAO B but also suppressed the MPP^+^-enhanced asparagine endopeptidase activity and alpha-synuclein N103 cleavage and decreased the full-length synuclein and cleaved synuclein levels [116]. The neurorescue effect of rasagiline in an MPTP mouse model was manifested in the activation of the Ras-dependent PI3K-Akt survival pathway [136].

MAO A knockdown and MAO B silencing influenced gene expression induced by rasagiline or selegiline in some studied cells (SH-SY5Y and U118MG) [199].

The protective action of some other MAO inhibitors is mainly related to MAO-independent effects. For example, the reversible MAO A inhibitors, the natural alkaloid beta-carbolines (harmaline, harmalol, and harmine), can act as ROS scavengers. Certain evidence exists that these effects may be attributable to their antimutagenic and antigenotoxic effects. Their antimutagenic activity was assayed in *Saccharomyces cerevisiae*, and the antigenotoxicity was tested by the comet assay in a V79 cell line [200]. The alkaloids had a significant protective effect against paraquat [200]. Beta-carbolines prevented the loss of cell viability in PC12 cells treated with MPP^+^, reduced the condensation and fragmentation of nuclei, and inhibited the decrease in mitochondrial transmembrane potential, cytochrome *c* release, activation of caspase-3, ROS formation, and depletion of GSH caused by MPP^+^ [143]. The compounds prevented mouse brain mitochondrial damage and PC12 cell death due to their scavenging action on ROS and thiol oxidation [142].

In an MPTP mouse model, MAO B inhibitor MT-20R prevented mitochondrial caspase-dependent apoptosis regulated by Bcl-2/Bax (it enhanced the expression of anti-apoptotic protein Bcl-2, decreased the expression of proapoptotic Bax and Caspase 3, and activated the PI3K/Akt/Nrf2/HO-1 signaling pathway) [139].

The positive effect of VAR, the iron-chelating inhibitor of MAO A and B, on motor impairments, the loss of striatal DA, and the decrease in serotonin levels in rats in 6-OHDA models extends beyond MAO inhibition and is probably associated with the iron-chelating properties [99].

The protective effect of isatin in MPTP and rotenone PD models is apparently realized in different ways. In the case of the MPTP-induced mouse model, the effect of isatin is primarily due to the inhibition of the activity of MAO B, which is responsible for the bioactivation of the MPTP into the neurotoxin MPP^+^ [147]. The effect on numerous isatin-binding proteins triggers various protective reactions, particularly by acting on the ubiquitin-proteasome system, which is involved not only in the elimination of proteins, but in the regulation of a wide variety of cellular processes, including genome stability, immune response, signal transduction, and much more [201,202]. Proteomic profiling of isatin-binding proteins in the brain revealed enzymes directly related to the ubiquitin-proteasome system: E3 ubiquitin protein ligase MYCBP2, ubiquitin-carboxyl-terminal hydrolase 24, E3 ubiquitin protein ligase MIB2, E3 ubiquitin protein ligase HUWE1, ubiquitin-conjugating enzyme variant 1, and polyubiquitin [203]. The effect of MPTP and/or isatin changed the mitochondrial subproteomes of the proteins interacting with the components of the Rpn10 and Rpn13 subunits of the proteasome regulatory subparticle [117,147].

In the case of the rotenone rat model of PD, the action of isatin is realized through the involvement of other isatin-binding proteins. This may be due to interspecies characteristics (the effects of isatin in mice and rats do not coincide [155]). On the other hand, this may depend on the obvious “wave-like” change in the level of target isatin-binding proteins. They change differently in the dynamics of PD development under the influence of both the neurotoxin and isatin itself, which is known to affect the relative level of many important brain proteins [153].

## 6. Conclusions

**Figure 2:**
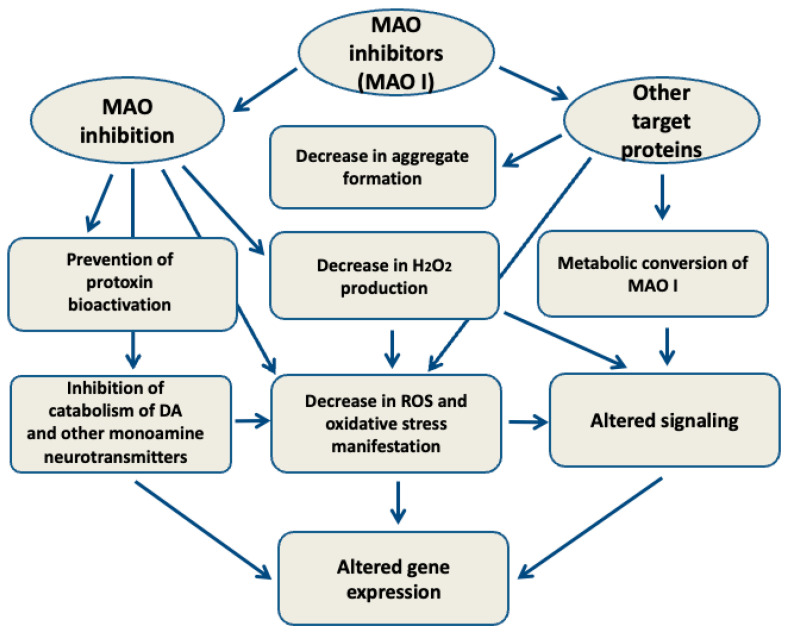
MAO-dependent and MAO-independent effects of MAO inhibitors. See explanations in the text. DA—dopamine; ROS—reactive oxygen species.

Numerous studies employing various animal and cell toxic models of PD have convincingly demonstrated a significant positive impact of MAO inhibitors, which can act in both a MAO-dependent and a MAO-independent manner (Figure 2).

In some cases (e.g., MPTP-induced PD) MAO B inhibitors prevent toxin bioactivation. Regardless of toxin type, the potent inhibition of MAO prevents DA loss, the formation of hydrogen peroxide, hydrogen peroxide signaling, and the accumulation of hydrogen peroxide-derived ROS, causing oxidative stress development, which involves altered gene expression. However, these effects related to MAO inhibition represent only the MAO-dependent part of the pharmacological activity associated with MAO inhibitors. Increasing evidence exists that some metabolites of MAO inhibitors (e.g., the rasagiline metabolite 1-R-aminoindan) possess bio-pharmacological activities unrelated to the parent compound. In addition, various MAO inhibitors exhibit multitarget action, in which MAO-independent effects prevail. This opens new prospects in the development of novel therapeutics based on simultaneous actions on several perspective targets for the therapy of PD.

In contrast to the well-documented positive impact of MAO inhibitors in neurotoxin- and pesticide-based models of PD, it still remains unclear whether they would be effective in the endotoxin (LPS)-based model of PD. Although the existing literature does not pay much attention to this problem [204], the anti-inflammatory therapeutic potential of MAO inhibitors [205,206,207] suggests that they would be effective in treating neuroinflammation, which is linked to PD.

## Figures and Tables

**Table 1:** MAO inhibitors in 6-OHDA models of Parkinson’s disease.

Inhibitor	Type of Inhibition	Model	Effects	References
L-Deprenyl (selegiline, *N*-methyl-1-phenyl-*N*-prop-2-ynylpropan-2-amine)	Selective irreversible MAO B inhibitor	Rats	Protection of sympathetic ganglion cell bodies and peripheral noradrenergic innervation.	[79,80]
L-Deprenyl (selegiline, *N*-methyl-1-phenyl-*N*-prop-2-ynylpropan-2-amine)	Selective irreversible MAO B inhibitor	Rats	Amelioration of effects on motor complications, induced by levodopa, and expression of proteins involved in these complications.	[81]
L-Deprenyl (selegiline, *N*-methyl-1-phenyl-*N*-prop-2-ynylpropan-2-amine)	Selective irreversible MAO B inhibitor	Rats	Deprenyl, co-administered with levodopa, did not influence behavioral recovery induced by fetal ventral mesencephalic grafts.	[82]
Clorgyline (N-[3-(2,4-dichlorophenoxy)propyl]-N-methyl-prop-2-yn-1-amine) L-Deprenyl (selegiline, *N*-methyl-1-phenyl-*N*-prop-2-ynylpropan-2-amine) or Rasagiline ((1R)-N-prop-2-ynyl-2,3-dihydro-1H-inden-1-amine) or TVP-101 [2,3-dihydro-N-2-propynyl-1 H-inden-1-amine-(1 R)-hydrochloride] or Lazabemide (Ro 19-6327, N-(2-aminoethyl)-5-chloropyridine-2-carboxamide)	Selective irreversible MAO A inhibitor Selective irreversible MAO B inhibitors Selective reversible MAO B inhibitors	Rats	Inhibition of glial MAO B increased local DA levels at the presynaptic receptors and reduced DA release by presynaptic inhibition. Inhibition of MAO A or MAO B reduced oxidative stress. Rasagiline exhibited an additional antioxidant effect independently of MAO inhibition.	[73,74,75,83]
Rasagiline ((1R)-N-prop-2-ynyl-2,3-dihydro-1H-inden-1-amine)	Selective irreversible MAO B inhibitors	Zebrafish	Prevented locomotor impairments and neuronal loss.	[84]
Rasagiline ((1R)-N-prop-2-ynyl-2,3-dihydro-1H-inden-1-amine)	Selective irreversible MAO B inhibitor	Rats	Increased the survival of dopaminergic neurons in the SN, abolished the motor stereotypies associated with nigrostriatal lesion.	[85]
Rasagiline 1-R-aminoindan, the major metabolite of rasagiline, and hydroxyaminoindan, metabolite of ladostigil ([(3R)-3-(prop-2-ynylamino)-2,3-dihydro-1H-inden-5-yl] N-ethyl-N-methylcarbamate)	Selective irreversible MAO B inhibitor	Rats with 6-OHDA and neurotoxin DSP-4	Increased levels of brain-derived neurotrophic factor (BDNF) in the hippocampus and striatum and sparing in the mitochondrial marker Hsp60 and tyrosine hydroxylase (TH) immunoreactive terminals in the striatum, hippocampus, and SN.	[86]
1-R-aminoindan, the major metabolite of rasagiline, and hydroxyaminoindan, metabolite of ladostigil ([(3R)-3-(prop-2-ynylamino)-2,3-dihydro-1H-inden-5-yl] N-ethyl-N-methylcarbamate)	Rasagiline metabolite	Rats	Normalized motor impairments and prevented the decrease in the DA, 3,4-dihydroxyphenylacetic acid (DOPAC), and homovanillic acid (HVA) levels in the striatum.	[87]
1-R-aminoindan, the major metabolite of rasagiline, and hydroxyaminoindan, metabolite of ladostigil ([(3R)-3-(prop-2-ynylamino)-2,3-dihydro-1H-inden-5-yl] N-ethyl-N-methylcarbamate)	Ladostigil exhibits irreversible MAO A and B inhibitory activity and acetylcholine-butyrylcholine esterase inhibitory activity [88]	PC12 cells	Pre-treatment with aminoindan or hydroxyaminoindan significantly increased the viability of the cells. These compounds did not show neurotoxic effects.	[87]
1-R-aminoindan, the major metabolite of rasagiline, and hydroxyaminoindan, metabolite of ladostigil ([(3R)-3-(prop-2-ynylamino)-2,3-dihydro-1H-inden-5-yl] N-ethyl-N-methylcarbamate)	Metabolite of rasagiline, selective irreversible MAO B inhibitor	Rats	Aminoindan restored motor impairments and significantly prevented the decline in striatal levels of DA, DOPAC, and homovanillic acid (HVA).	[89,90]
Beta-carbolines: harmaline (7-methoxy-1-methyl-4,9-dihydro-3H-pyrido [3,4-b]indole), harmalol (1-methyl-4,9-dihydro-3H-pyrido[3,4-b]indol-7-ol), and harmine (7-methoxy-1-methyl-9H-pyrido[3,4-b]indole)	Reversible MAO A inhibitors	Rat brain mitochondria and synaptosomes	Protection against oxidative damage, mitochondrial swelling. and membrane potential loss. Decrease in synaptosomal calcium uptake, prevention of catecholamine-induced thioredoxin reductase inhibition, thiol oxidation, and carbonyl formation in mitochondria and synaptosomes; decrease in ROS-induced deoxyribose degradation.	[91]
Beta-carbolines (harmaline, harmalol and harmine)	Reversible MAO A inhibitors	PC12 cells	Beta-carbolines attenuated the loss of cell viability. Harmaline and harmalol reduced the catecholamine-induced membrane potential loss.	[91]
Moclobemide (4-chloro-*N*-(2-morpholin-4-ylethyl)benzamide)	Reversible MAO A inhibitor	Rats	Increase in contraversive rotational behavior only in case of co-administration with levodopa.	[79]
Afobazole 4-[2-[(6-ethoxy-1*H*-benzimidazol-2-yl)sulfanyl]ethyl]morpholine	Reversible MAO A inhibitor	Mice	Normalized motor dysfunction, restored the DA level in the striatum and did not affect the contents of norepinephrine, serotonin or its metabolites.	[92,93]
Lazabemide (N-(2-aminoethyl)-5-chloropyridine-2-carboxamide)	Reversible MAO B inhibitor	Rats	Increase in contraversive rotational behavior only in case of co-administration with levodopa.	[79]
PF 9601N (N-[(5-phenylmethoxy-1H-indol-2-yl)methyl]prop-2-yn-1-amine)	Selective reversible MAO B inhibitor	Rats	Decreased the loss of tyrosine hydroxylase positive neurons in the SN. Reduced 6-OHDA-induced neurodegeneration.	[94]
Safinamide ((2S)-2-[[4-[(3-fluorophenyl) methoxy]phenyl] methylamino] propanamide)	Selective reversible MAO B inhibitor, sodium channel blocker	Rats	Prevention of the levodopa-induced increase in striatal glutamate associated with dyskinesia appearance. Suppression of microglial activation and protection of DA neurons in the SN from degeneration. Reduction in the firing rate and the synaptic currents of striatal projection neurons.	[95,96,97]
(Hetero)arylalkenylpropargyl amines (especially compound1, the m-fluorophenyl compound 24, the m-benzyloxyphenyl compound 31, 3,4-dimethylphenyl compound 45, 3,4-difluorophenyl compound 46, and the 3- methyl-4-fluorophenyl analogue 48)	MAO B irreversible inhibitors	PC12 cells	Neuroprotective properties in vitro.	[98]
VAR (5-[2-(methyl-prop-2-ynyl-amino)- ethyl]-quinolin-8-ol dihydrochloride)	Iron-chelating MAO A and B inhibitor	Rats	Attenuation of motor impairments and significant reduction in the striatal DA loss. Increase in 5HT levels in the striatum and hippocampus.	[99]

**Table 2:** MAO inhibitors in MPTP models of Parkinson’s disease.

Inhibitor	Type of Inhibition	Model	Effects	References
Pargyline (N-benzyl-N- methylprop-2-yn-1-amine)	Non-selective irreversible MAO inhibitor	Non-human primates	Protection against nigrostriatal DA neurotoxicity, reduction in brain MPP^+^ levels.	[127]
Clorgyline (N-[3-(2,4-dichlorophenoxy) propyl]-N- methyl-prop-2-yn-1-amine)	Selective irreversible MAO A inhibitor	Goldfish	Lack of protection against loss of movement.	[128]
L-Deprenyl (selegiline, *N*- methyl-1-phenyl-*N*-prop-2-ynylpropan-2-amine)	Selective irreversible MAO B inhibitor	Rats	Neuroprotection; inhibition of hydroxyl radical formation and restoration of striatal DA levels.	[129]
L-Deprenyl (selegiline, *N*- methyl-1-phenyl-*N*-prop-2-ynylpropan-2-amine)	Selective irreversible MAO B inhibitor	Goldfish	Protection from loss of movement.	[128]
L-Deprenyl (selegiline, *N*- methyl-1-phenyl-*N*-prop-2-ynylpropan-2-amine) SH-SY5Y cells	Selective irreversible MAO B inhibitor	Primary neuronal cultures of mouse midbrain DA neurons	MAO B-independent increase in expression of thioredoxin, manganese superoxide dismutase, and antiapoptotic Bcl-2, supporting cell survival.	[130]
L-Deprenyl (selegiline, *N*- methyl-1-phenyl-*N*-prop-2-ynylpropan-2-amine)	Selective irreversible MAO B inhibitor	SH-SY5Y cells	Attenuation of the MPTP-induced autophagic response and protection against cell death.	[113]
L-Deprenyl (selegiline, *N*- methyl-1-phenyl-*N*-prop-2-ynylpropan-2-amine) Pargyline (N-benzyl-N- methylprop-2-yn-1-amine) Nialamide (N-benzyl-3-[2-(pyridine-4-carbonyl) hydrazinyl] propanamide) Tranylcypromine ((1R,2S)-2-phenylcyclopropan-1-amine)	Selective irreversible MAO B inhibitor Non-selective irreversible MAO inhibitors	Mice	All the inhibitors effectively protected against the nigrostriatal DA neurotoxicity of MPTP and prevented the neostriatal DA loss.	[131]
Rasagiline ((1R)-N-prop-2-ynyl-2,3- dihydro-1H-inden-1-amine) L-Deprenyl (selegiline, *N*- methyl-1-phenyl-*N*-prop-2-ynylpropan-2-amine)	Selective irreversible MAO B inhibitors Selective irreversible MAO B inhibitor	Non-human primates	Both inhibitors restored motor impairments, the number of DA cells in the SN, and striatal DA levels.	[132]
Rasagiline (Hetero)arylalkenylpropargylamines SZV558 (methyl-(2-phenyl-allyl)-prop-2-ynyl-amine hydrochloride) and SZV2220	MAO B irreversible inhibitors	Mice	Restored locomotor activity, DA, and its metabolite content in the striatum. SZV558 expressed the highest neuroprotective action.	[133]
Rasagiline ((1R)-N-prop-2-ynyl-2,3-dihydro-1H-inden-1-amine)	MAO B irreversible inhibitor	SH-SY5Y cells Mice	Decrease in the MPP+-enhanced asparagine endopeptidase activity and alpha-synuclein N103 cleavage.	[116]
Rasagiline ((1R)-N-prop-2-ynyl-2,3-dihydro-1H-inden-1-amine)	Selective irreversible MAO B inhibitor [134]	Mice	Restoration of dopaminergic cell reduction, striatal DA, and TH. Activation of cell signaling survival cascades (Trk, Ras-PI3K-Akt, and others).	[135,136]
M30 [5-(*N*-methyl-*N*-propargyl- amino-methyl)-8-hydroxyquinone]	Brain-permeable MAO A/B inhibitor with iron-chelating activity.	Mice	Elevation of striatal DA, 5HT, and noradrenaline levels, TH protein level and activity. Increase in dopaminergic and transferrin receptor cells in the SN and in hypoxia-induced factor (HIF).	[137,138]
MT-20R (a derivative of ladostigil, [(3R)-3-(prop-2-ynylamino) indan-5-yl]-N-propylcarbamate)	MAO B inhibitor	Mice	Alleviation of motor deficits, increase in the level of DA and its metabolites, restoration of TH expression and the number of TH-positive neurons in the SN. Increase in the expression of Bcl-2, decrease in the expression of Bax and Caspase 3, and activation of the AKT/Nrf2/HO-1 signaling pathway.	[139]
VAR (5-[2-(methyl-prop-2-ynyl-amino)- ethyl]-quinolin-8-ol dihydrochloride)	Iron-chelating MAO A and B inhibitor	Mice	Attenuation of motor impairments, prevention of striatal DA loss, increase in 5HT levels in the striatum and hippocampus, and increase in the TH level.	[99]
Lamotrigine (6-(2,3-dichlorophenyl)-1,2,4-triazine-3,5-diamine)	MAO B inhibitor [140]	Mice	Protection against DA neuronal death in the SN, promotion of striatal dendrite sprouting, maintenance of high levels of the DA transporter, TH immunoreactive neurons, and DA content.	[141]
Beta-carbolines (harmaline, harmalol, and harmine)	Reversible MAO A inhibitors	Mice	Harmalol reduced the MPTP effect on the enzyme activities and formation of tissue peroxidation products. Harmaline, harmalol, and harmine attenuated the MPP^+^-induced inhibition of electron flow and membrane potential formation and the DA-induced thiol oxidation and carbonyl formation in mitochondria.	[142]
Beta-carbolines (harmaline, harmalol, and harmine)	Reversible MAO A inhibitors	PC12 cells	Prevented the loss of viability of MPP^+^-treated cells, reduced condensation and fragmentation of nuclei, inhibited the decrease in mitochondrial membrane potential, cytochrome *c* release, activation of caspase-3, ROS formation, and depletion of GSH.	[143]
Curcumin ((1E,6E)-1,7-bis(4-hydroxy-3-methoxyphenyl) hepta-1,6-diene-3,5-dione) and its metabolite tetrahydrocurcumin (1,7-bis(4-hydroxy-3-methoxyphenyl) heptane- 3,5-dione)	MAO B inhibitors	Mice	Neuroprotection against MPTP-induced neurotoxicity: reversion of MPTP-induced depletion of DA and DOPAC.	[144]
Isatin (indoledione-2,3)	MAO B inhibitor	Mice	Reduced motor manifestations of MPTP-induced neurotoxicity, influenced the profiles of numerous brain isatin-binding proteins.	[117,118,145,146,147]
Phenelzine (2-phenylethylhydrazine)	Non-selective irreversible MAO inhibitor	PC12 cells	Attenuation in the cell viability loss. Reduction in condensation and fragmentation of nuclei, prevention of the decrease in mitochondrial membrane potential, release of cytochrome *c*, ROS formation, and glutathione depletion.	[148]
Catalpol ((2S,3R,4S,5S,6R)-2-[[(1S,2S,4S,5S,6R,10S)-5-hydroxy-2-(hydroxymethyl)-3,9-dioxatricyclo[4.4.0.02,4]dec-7-en-10-yl]oxy]-6-(hydroxymethyl)oxane-3,4,5-triol)	MAO B inhibitor, an iridoid glycoside present in the roots of Rehmannia glutinosa, the traditional Chinese medicinal herb [149]	Mice	Restoration of locomotor ability, increase in striatal DA levels without changing the metabolite/DA ratios, increase in the TH-positive neurons, striatal DA transporter, and the striatal glial cell-derived neurotrophic factor protein level. Elevation of the expression of striatal glial cell line-derived neurotrophic factor.	[150]
Catalpol ((2S,3R,4S,5S,6R)-2-[[(1S,2S,4S,5S,6R,10S)-5-hydroxy-2-(hydroxymethyl)-3,9-dioxatricyclo[4.4.0.02,4]dec-7-en-10-yl]oxy]-6-(hydroxymethyl)oxane-3,4,5-triol)	MAO B inhibitor present in roots of Rehmannia glutinosa	Astrocytes	Attenuation of mitochondrial dysfunction by reversing the activity of complex I, membrane potential, intracellular Ca^2+^ level, and ROS accumulation.	[151]
Catalpol ((2S,3R,4S,5S,6R)-2-[[(1S,2S,4S,5S,6R,10S)-5-hydroxy-2-(hydroxymethyl)-3,9-dioxatricyclo[4.4.0.02,4]dec-7-en-10-yl]oxy]-6-(hydroxymethyl)oxane-3,4,5-triol)	MAO B inhibitor	Cultured mesencephalic neurons	Increase in neuron viability and prevention of DA neuron death, inhibition of mitochondrial complex I, and the loss of mitochondrial membrane potential. Reduction in lipid peroxidation and increase in the activity of glutathione peroxidase and superoxide dismutase.	[152]

**Table 3:** MAO inhibitors in rotenone and paraquat models of Parkinson’s disease.

Inhibitor	Type of Inhibition	Model, Toxin	Effects	References
L-Deprenyl (eldepryl, selegiline, *N*-methyl-1-phenyl-*N*-prop-2-ynylpropan-2-amine)	Selective irreversible MAO B inhibitor	Rats **Rotenone**	Inhibition of stereotypic rotations, restoration of complex I activity and glutathione levels in SN, TH immunoreactivity, and striatal DA. Increase in activities of superoxide dismutase and catalase.	[181]
L-Deprenyl (eldepryl, selegiline, *N*-methyl-1-phenyl-*N*-prop-2-ynylpropan-2-amine)	Selective irreversible MAO B inhibitor	Rats **Rotenone**	Decrease in the numbers of glial fibrillary acidic protein (GFAP)- and integrin alphaM (CD11b)-positive cells and expression of GFAP and CD11b in SN and striatum. Prevention of expression of Beclin1 and microtubule-associated protein 1 light chain 3 (LC3) in SN.	[182,183]
L-Deprenyl (eldepryl, selegiline, *N*-methyl-1-phenyl-*N*-prop-2-ynylpropan-2-amine)	Selective irreversible MAO B inhibitor	Rats **Paraquat**	Restoration of locomotor activity and increase in the striatal DA level.	[184]
(Hetero) arylalkenylpropargylamines (compound 1, the m-fluorophenyl compound 24, the m-benzyloxyphenyl compound 31, 3,4-dimethylphenyl compound 45, 3,4-difluorophenyl compound 46, and the 3- methyl-4-fluorophenyl analogue 48)	MAO B irreversible inhibitors	PC-12 cells **Rotenone**	In vitro neuroprotective properties.	[98]
(Hetero) arylalkenylpropargylamines SZV558	MAO B irreversible inhibitors	Slices of rat striatum **Rotenone**	The compounds (especially SZV558) exhibited protective effects against pathological DA release and formation of toxic DA quinone and rescued TH positive neurons in SN.	[133]
Rasagiline	MAO B irreversible inhibitor	SHSY5Y cells **Paraquat**	Protection against cell death by reducing caspase 3 activation, ROS generation, and the fall in mitochondrial membrane potential. Increase in cellular glutathione levels.	[185]
Isatin (indoledione-2,3) Afobazole	MAO B inhibitor MAO A inhibitor	Rats **Rotenone**	Restoration of locomotor activity. Altered relative content of proteins associated with neurodegeneration (e.g., synuclein, DJ-1, GAPDH, TRIM2, E3-ubiquitin ligase Tripartite motif-containing protein 2, Prohibitin-2).	[186,187] [186]

## References

[1] Ben-Shlomo Y., Darweesh S., Llibre-Guerra J., Marras C., San Luciano M., Tanner C. (2024). The Epidemiology of Parkinson’s Disease. Lancet.

[2] Costa H.N., Esteves A.R., Empadinhas N., Cardoso S.M. (2022). Parkinson’s Disease: A Multisystem Disorder. Neurosci. Bull..

[3] Dauer W., Przedborski S. (2003). Parkinson’s Disease: Mechanisms and Models. Neuron.

[4] Feigin V.L., Nichols E., Alam T., Bannick M.S., Beghi E., Blake N., Culpepper W.J., Dorsey E.R., Elbaz A., Ellenbogen R.G. (2019). Global, Regional, and National Burden of Neurological Disorders, 1990–2016: A Systematic Analysis for the Global Burden of Disease Study 2016. Lancet Neurol..

[5] Kouli A., Torsney K.M., Kuan W.-L., Stoker T.B., Greenland J.C. (2018). Parkinson’s Disease: Etiology, Neuropathology, and Pathogenesis. Parkinson’s Disease: Pathogenesis and Clinical Aspects.

[6] Armstrong M.J., Okun M.S. (2020). Diagnosis and Treatment of Parkinson Disease: A Review. JAMA.

[7] Baggett D., Olson A., Parmar M.S. (2024). Novel Approaches Targeting α-Synuclein for Parkinson’s Disease: Current Progress and Future Directions for the Disease-Modifying Therapies. Brain Disord..

[8] Morris H.R., Spillantini M.G., Sue C.M., Williams-Gray C.H. (2024). The Pathogenesis of Parkinson’s Disease. Lancet.

[9] Oliveira L.M.A., Gasser T., Edwards R., Zweckstetter M., Melki R., Stefanis L., Lashuel H.A., Sulzer D., Vekrellis K., Halliday G.M. (2021). Alpha-Synuclein Research: Defining Strategic Moves in the Battle against Parkinson’s Disease. NPJ Park. Dis..

[10] Poewe W., Seppi K., Tanner C.M., Halliday G.M., Brundin P., Volkmann J., Schrag A.-E., Lang A.E. (2017). Parkinson Disease. Nat. Rev. Dis. Primers.

[11] Sulzer D., Edwards R.H. (2019). The Physiological Role of α-Synuclein and Its Relationship to Parkinson’s Disease. J. Neurochem..

[12] Adler C.H., Beach T.G. (2016). Neuropathological Basis of Nonmotor Manifestations of Parkinson’s Disease. Mov. Disord..

[13] Marras C., Chaudhuri K.R. (2016). Nonmotor Features of Parkinson’s Disease Subtypes. Mov. Disord..

[14] Ye H., Robak L.A., Yu M., Cykowski M., Shulman J.M. (2023). Genetics and Pathogenesis of Parkinson’s Syndrome. Annu. Rev. Pathol..

[15] Aryal B., Lee Y. (2019). Disease Model Organism for Parkinson Disease: Drosophila Melanogaster. BMB Rep..

[16] Koprich J.B., Kalia L.V., Brotchie J.M. (2017). Animal Models of α-Synucleinopathy for Parkinson Disease Drug Development. Nat. Rev. Neurosci..

[17] Li H., Ham A., Ma T.C., Kuo S.-H., Kanter E., Kim D., Ko H.S., Quan Y., Sardi S.P., Li A. (2019). Mitochondrial Dysfunction and Mitophagy Defect Triggered by Heterozygous GBA Mutations. Autophagy.

[18] Rahul, Siddique Y.H. (2022). Drosophila: A Model to Study the Pathogenesis of Parkinson’s Disease. CNS Neurol. Disord. Drug Targets.

[19] Ramonet D., Daher J.P.L., Lin B.M., Stafa K., Kim J., Banerjee R., Westerlund M., Pletnikova O., Glauser L., Yang L. (2011). Dopaminergic Neuronal Loss, Reduced Neurite Complexity and Autophagic Abnormalities in Transgenic Mice Expressing G2019S Mutant LRRK2. PLoS ONE.

[20] Falkenburger B.H., Schulz J.B., Riederer P., Reichmann H., Youdim M.B.H., Gerlach M. (2006). Limitations of Cellular Models in Parkinson’s Disease Research. Parkinson’s Disease and Related Disorders.

[21] Ioghen O.C., Ceafalan L.C., Popescu B.O. (2023). SH-SY5Y Cell Line In Vitro Models for Parkinson Disease Research-Old Practice for New Trends. J. Integr. Neurosci..

[22] Weykopf B., Haupt S., Jungverdorben J., Flitsch L.J., Hebisch M., Liu G.-H., Suzuki K., Belmonte J.C.I., Peitz M., Blaess S. (2019). Induced Pluripotent Stem Cell-Based Modeling of Mutant LRRK2-Associated Parkinson’s Disease. Eur. J. Neurosci..

[23] Winner B., Jappelli R., Maji S.K., Desplats P.A., Boyer L., Aigner S., Hetzer C., Loher T., Vilar M., Campioni S. (2011). In Vivo Demonstration That Alpha-Synuclein Oligomers Are Toxic. Proc. Natl. Acad. Sci. USA.

[24] Ke M., Chong C.-M., Zhu Q., Zhang K., Cai C.-Z., Lu J.-H., Qin D., Su H. (2021). Comprehensive Perspectives on Experimental Models for Parkinson’s Disease. Aging Dis..

[25] Konnova E.A., Swanberg M., Stoker T.B., Greenland J.C. (2018). Animal Models of Parkinson’s Disease. Parkinson’s Disease: Pathogenesis and Clinical Aspects.

[26] Lama J., Buhidma Y., Fletcher E.J.R., Duty S. (2021). Animal Models of Parkinson’s Disease: A Guide to Selecting the Optimal Model for Your Research. Neuronal Signal..

[27] Zeng X.-S., Geng W.-S., Jia J.-J. (2018). Neurotoxin-Induced Animal Models of Parkinson Disease: Pathogenic Mechanism and Assessment. ASN Neuro.

[28] Blesa J., Phani S., Jackson-Lewis V., Przedborski S. (2012). Classic and New Animal Models of Parkinson’s Disease. J. Biomed. Biotechnol..

[29] Khan E., Hasan I., Haque M.E. (2023). Parkinson’s Disease: Exploring Different Animal Model Systems. Int. J. Mol. Sci..

[30] Naoi M., Maruyama W., Shamoto-Nagai M., Riederer P. (2024). Toxic Interactions between Dopamine, α-Synuclein, Monoamine Oxidase, and Genes in Mitochondria of Parkinson’s Disease. J. Neural Transm..

[31] Yeung A.W.K., Georgieva M.G., Atanasov A.G., Tzvetkov N.T. (2019). Monoamine Oxidases (MAOs) as Privileged Molecular Targets in Neuroscience: Research Literature Analysis. Front. Mol. Neurosci..

[32] Youdim M.B.H., Edmondson D., Tipton K.F. (2006). The Therapeutic Potential of Monoamine Oxidase Inhibitors. Nat. Rev. Neurosci..

[33] Shih J.C., Chen K., Ridd M.J. (1999). Monoamine Oxidase: From Genes to Behavior. Annu. Rev. Neurosci..

[34] Shih J.C., Wu J.B., Chen K. (2011). Transcriptional Regulation and Multiple Functions of MAO Genes. J. Neural Transm..

[35] Finberg J.P.M. (2014). Update on the Pharmacology of Selective Inhibitors of MAO-A and MAO-B: Focus on Modulation of CNS Monoamine Neurotransmitter Release. Pharmacol. Ther..

[36] Shih J.C. (2018). Monoamine oxidase isoenzymes: Genes, functions and targets for behavior and cancer therapy. J. Neural Transm..

[37] Westlund K.N., Denney R.M., Rose R.M., Abell C.W. (1988). Localization of Distinct Monoamine Oxidase A and Monoamine Oxidase B Cell Populations in Human Brainstem. Neuroscience.

[38] Finberg J.P.M., Rabey J.M. (2016). Inhibitors of MAO-A and MAO-B in Psychiatry and Neurology. Front. Pharmacol..

[39] Naoi M., Maruyama W., Inaba-Hasegawa K. (2012). Type A and B Monoamine Oxidase in Age-Related Neurodegenerative Disorders: Their Distinct Roles in Neuronal Death and Survival. Curr. Top. Med. Chem..

[40] Reyes-Chaparro A., Flores-Lopez N.S., Quintanilla-Guerrero F., Nicolás-Álvarez D.E., Hernandez-Martinez A.R. (2023). Design of New Reversible and Selective Inhibitors of Monoamine Oxidase A and a Comparison with Drugs Already Approved. Bull. Natl. Res. Cent..

[41] Naoi M., Maruyama W. (2009). Functional Mechanism of Neuroprotection by Inhibitors of Type B Monoamine Oxidase in Parkinson’s Disease. Expert. Rev. Neurother..

[42] Nakamura Y., Arawaka S., Sato H., Sasaki A., Shigekiyo T., Takahata K., Tsunekawa H., Kato T. (2021). Monoamine Oxidase-B Inhibition Facilitates α-Synuclein Secretion In Vitro and Delays Its Aggregation in rAAV-Based Rat Models of Parkinson’s Disease. J. Neurosci..

[43] Alborghetti M., Bianchini E., De Carolis L., Galli S., Pontieri F.E., Rinaldi D. (2024). Type-B Monoamine Oxidase Inhibitors in Neurological Diseases: Clinical Applications Based on Preclinical Findings. Neural Regen. Res..

[44] Carradori S., Secci D., Petzer J.P. (2018). MAO Inhibitors and Their Wider Applications: A Patent Review. Expert. Opin. Ther. Pat..

[45] Youdim M.B.H. (2018). Monoamine Oxidase Inhibitors, and Iron Chelators in Depressive Illness and Neurodegenerative Diseases. J. Neural Transm..

[46] Blandini F., Armentero M.-T., Martignoni E. (2008). The 6-Hydroxydopamine Model: News from the Past. Park. Relat. Disord..

[47] Simola N., Morelli M., Carta A.R. (2007). The 6-Hydroxydopamine Model of Parkinson’s Disease. Neurotox. Res..

[48] Chotibut T., Apple D.M., Jefferis R., Salvatore M.F. (2012). Dopamine Transporter Loss in 6-OHDA Parkinson’s Model Is Unmet by Parallel Reduction in Dopamine Uptake. PLoS ONE.

[49] Storch A., Ludolph A.C., Schwarz J. (2004). Dopamine Transporter: Involvement in Selective Dopaminergic Neurotoxicity and Degeneration. J. Neural Transm..

[50] Varešlija D., Tipton K.F., Davey G.P., McDonald A.G. (2020). 6-Hydroxydopamine: A Far from Simple Neurotoxin. J. Neural Transm..

[51] Glinka Y.Y., Youdim M.B. (1995). Inhibition of Mitochondrial Complexes I and IV by 6-Hydroxydopamine. Eur. J. Pharmacol..

[52] Hernandez-Baltazar D., Zavala-Flores L.M., Villanueva-Olivo A. (2017). The 6-Hydroxydopamine Model and Parkinsonian Pathophysiology: Novel Findings in an Older Model. Neurología.

[53] Pantic I., Cumic J., Skodric S.R., Dugalic S., Brodski C. (2021). Oxidopamine and Oxidative Stress: Recent Advances in Experimental Physiology and Pharmacology. Chem.-Biol. Interact..

[54] Schober A. (2004). Classic Toxin-Induced Animal Models of Parkinson’s Disease: 6-OHDA and MPTP. Cell Tissue Res..

[55] Borisenko G.G., Kagan V.E., Hsia C.J.C., Schor N.F. (2000). Interaction between 6-Hydroxydopamine and Transferrin:  “Let My Iron Go”. Biochemistry.

[56] Jameson G.N.L., Jameson R.F., Linert W. (2004). New Insights into Iron Release from Ferritin: Direct Observation of the Neurotoxin 6-Hydroxydopamine Entering Ferritin and Reaching Redox Equilibrium with the Iron Core. Org. Biomol. Chem..

[57] Ben-Shachar D., Zuk R., Gazawi H., Ljubuncic P. (2004). Dopamine toxicity involves mitochondrial complex I inhibition: Implications to dopamine-related neuropsychiatric disorders. Biochem. Pharmacol..

[58] Hanrott K., Gudmunsen L., O’Neill M.J., Wonnacott S. (2006). 6-Hydroxydopamine-Induced Apoptosis Is Mediated via Extracellular Auto-Oxidation and Caspase 3-Dependent Activation of Protein Kinase Cδ. J. Biol. Chem..

[59] Saito Y., Nishio K., Ogawa Y., Kinumi T., Yoshida Y., Masuo Y., Niki E. (2007). Molecular Mechanisms of 6-Hydroxydopamine-Induced Cytotoxicity in PC12 Cells: Involvement of Hydrogen Peroxide-Dependent and -Independent Action. Free Radic. Biol. Med..

[60] Duan W.-J., Liang L., Pan M.-H., Lu D.-H., Wang T.-M., Li S.-B., Zhong H.-B., Yang X.-J., Cheng Y., Liu B. (2020). Theacrine, a Purine Alkaloid from Kucha, Protects against Parkinson’s Disease through SIRT3 Activation. Phytomedicine.

[61] Ramazani E., YazdFazeli M., Emami S.A., Mohtashami L., Javadi B., Asili J., Tayarani-Najaran Z. (2020). Protective Effects of Cinnamomum Verum, Cinnamomum Cassia and Cinnamaldehyde against 6-OHDA-Induced Apoptosis in PC12 Cells. Mol. Biol. Rep..

[62] Tai S., Zheng Q., Zhai S., Cai T., Xu L., Yang L., Jiao L., Zhang C. (2020). Alpha-Lipoic Acid Mediates Clearance of Iron Accumulation by Regulating Iron Metabolism in a Parkinson’s Disease Model Induced by 6-OHDA. Front. Neurosci..

[63] Zhai H., Kang Z., Zhang H., Ma J., Chen G. (2019). Baicalin Attenuated Substantia Nigra Neuronal Apoptosis in Parkinson’s Disease Rats via the mTOR/AKT/GSK-3β Pathway. JIN.

[64] Hayes J.P., Tipton K.F. (2002). Interactions of the Neurotoxin 6-Hydroxydopamine with Glyceraldehyde-3-Phosphate Dehydrogenase. Toxicol. Lett..

[65] Södersten E., Toskas K., Rraklli V., Tiklova K., Björklund Å.K., Ringnér M., Perlmann T., Holmberg J. (2018). A Comprehensive Map Coupling Histone Modifications with Gene Regulation in Adult Dopaminergic and Serotonergic Neurons. Nat. Commun..

[66] Lessner G., Schmitt O., Haas S.J.-P., Mikkat S., Kreutzer M., Wree A., Glocker M.O. (2010). Differential Proteome of the Striatum from Hemiparkinsonian Rats Displays Vivid Structural Remodeling Processes. J. Proteome Res..

[67] Park B., Yang J., Yun N., Choe K.-M., Jin B.K., Oh Y.J. (2010). Proteomic Analysis of Expression and Protein Interactions in a 6-Hydroxydopamine-Induced Rat Brain Lesion Model. Neurochem. Int..

[68] Andrew R., Watson D.G., Best S.A., Midgley J.M., Wenlong H., Petty R.K. (1993). The Determination of Hydroxydopamines and Other Trace Amines in the Urine of Parkinsonian Patients and Normal Controls. Neurochem. Res..

[69] Curtius H.C., Wolfensberger M., Steinmann B., Redweik U., Siegfried J. (1974). Mass Fragmentography of Dopamine and 6-Hydroxydopamine. Application to the Determination of Dopamine in Human Brain Biopsies from the Caudate Nucleus. J. Chromatogr..

[70] Borah A., Mohanakumar K.P. (2010). L-DOPA-Induced 6-Hydroxydopamine Production in the Striata of Rodents Is Sensitive to the Degree of Denervation. Neurochem. Int..

[71] Borah A., Mohanakumar K.P. (2010). Long Term L-DOPA Treatment Causes Production of 6-OHDA in the Mouse Striatum: Involvement of Hydroxyl Radical. Ann. Neurosci..

[72] Riederer P., Horowski R. (2023). L-DOPA-Therapy in Parkinson’s Disease: Some Personal Reflections on L-DOPA Therapy from Vienna and Berlin. J. Neural Transm..

[73] Finberg J.P., Wang J., Goldstein D.S., Kopin I.J., Bankiewicz K.S. (1995). Influence of Selective Inhibition of Monoamine Oxidase A or B on Striatal Metabolism of L-DOPA in Hemiparkinsonian Rats. J. Neurochem..

[74] Sader-Mazbar O., Loboda Y., Rabey M.J., Finberg J.P.M. (2013). Increased L-DOPA-Derived Dopamine Following Selective MAO-A or -B Inhibition in Rat Striatum Depleted of Dopaminergic and Serotonergic Innervation. Br. J. Pharmacol..

[75] Wachtel S.R., Abercrombie E.D. (1994). L-3,4-Dihydroxyphenylalanine-Induced Dopamine Release in the Striatum of Intact and 6-Hydroxydopamine-Treated Rats: Differential Effects of Monoamine Oxidase A and B Inhibitors. J. Neurochem..

[76] Breese G.R., Traylor T.D. (1970). Effect of 6-hydroxydopamine on brain norepinephrine and dopamine evidence for selective degeneration of catecholamine neurons. J. Pharmacol. Exp. Ther..

[77] Ambani L.M., Van Woert M.H., Murphy S. (1975). Brain Peroxidase and Catalase in Parkinson Disease. Arch. Neurol..

[78] Boll M.-C., Alcaraz-Zubeldia M., Montes S., Rios C. (2008). Free Copper, Ferroxidase and SOD1 Activities, Lipid Peroxidation and NO(x) Content in the CSF. A Different Marker Profile in Four Neurodegenerative Diseases. Neurochem. Res..

[79] MacInnes N., Duty S. (2004). Locomotor Effects of Imidazoline I2-Site-Specific Ligands and Monoamine Oxidase Inhibitors in Rats with a Unilateral 6-Hydroxydopamine Lesion of the Nigrostriatal Pathway. Br. J. Pharmacol..

[80] Salonen T., Haapalinna A., Heinonen E., Suhonen J., Hervonen A. (1996). Monoamine Oxidase B Inhibitor Selegiline Protects Young and Aged Rat Peripheral Sympathetic Neurons against 6-Hydroxydopamine-Induced Neurotoxicity. Acta Neuropathol..

[81] Tsunekawa H., Takahata K., Okano M., Ishikawa T., Satoyoshi H., Nishimura T., Hoshino N., Muraoka S. (2018). Selegiline Increases on Time without Exacerbation of Dyskinesia in 6-Hydroxydopamine-Lesioned Rats Displaying l-Dopa-Induced Wearing-off and Abnormal Involuntary Movements. Behav. Brain Res..

[82] Adams C.E., Hoffman A.F., Hudson J.L., Hoffer B.J., Boyson S.J. (1994). Chronic Treatment with Levodopa and/or Selegiline Does Not Affect Behavioral Recovery Induced by Fetal Ventral Mesencephalic Grafts in Unilaterally 6-Hydroxydopamine-Lesioned Rats. Exp. Neurol..

[83] Aluf Y., Vaya J., Khatib S., Loboda Y., Finberg J.P.M. (2013). Selective Inhibition of Monoamine Oxidase A or B Reduces Striatal Oxidative Stress in Rats with Partial Depletion of the Nigro-Striatal Dopaminergic Pathway. Neuropharmacology.

[84] Cronin A., Grealy M. (2017). Neuroprotective and Neuro-Restorative Effects of Minocycline and Rasagiline in a Zebrafish 6-Hydroxydopamine Model of Parkinson’s Disease. Neuroscience.

[85] Blandini F., Armentero M.T., Fancellu R., Blaugrund E., Nappi G. (2004). Neuroprotective Effect of Rasagiline in a Rodent Model of Parkinson’s Disease. Exp. Neurol..

[86] Ledreux A., Boger H.A., Hinson V.K., Cantwell K., Granholm A.-C. (2016). BDNF Levels Are Increased by Aminoindan and Rasagiline in a Double Lesion Model of Parkinson’s Disease. Brain Res..

[87] Bar-Am O., Amit T., Youdim M.B.H. (2007). Aminoindan and Hydroxyaminoindan, Metabolites of Rasagiline and Ladostigil, Respectively, Exert Neuroprotective Properties in Vitro. J. Neurochem..

[88] Weinreb O., Amit T., Bar-Am O., Youdim M.B.H. (2012). Ladostigil: A Novel Multimodal Neuroprotective Drug with Cholinesterase and Brain-Selective Monoamine Oxidase Inhibitory Activities for Alzheimer’s Disease Treatment. Curr. Drug Targets.

[89] Weinreb O., Amit T., Sagi Y., Drigues N., Youdim M.B.H. (2009). Genomic and Proteomic Study to Survey the Mechanism of Action of the Anti-Parkinson’s Disease Drug, Rasagiline Compared with Selegiline, in the Rat Midbrain. J. Neural Transm..

[90] Weinreb O., Bar-Am O., Prosolovich K., Amit T., Youdim M.B.H. (2011). Does 1-(R)-Aminoindan Possess Neuroprotective Properties against Experimental Parkinson’s Disease?. Antioxid. Redox Signal..

[91] Kim D.H., Jang Y.Y., Han E.S., Lee C.S. (2001). Protective Effect of Harmaline and Harmalol against Dopamine- and 6-Hydroxydopamine-Induced Oxidative Damage of Brain Mitochondria and Synaptosomes, and Viability Loss of PC12 Cells. Eur. J. Neurosci..

[92] Kadnikov I.A., Verbovaya E.R., Voronkov D.N., Voronin M.V., Seredenin S.B. (2020). Deferred Administration of Afobazole Induces Sigma1R-Dependent Restoration of Striatal Dopamine Content in a Mouse Model of Parkinson’s Disease. Int. J. Mol. Sci..

[93] Voronin M.V., Kadnikov I.A., Voronkov D.N., Seredenin S.B. (2019). Chaperone Sigma1R Mediates the Neuroprotective Action of Afobazole in the 6-OHDA Model of Parkinson’s Disease. Sci. Rep..

[94] Cutillas B., Ambrosio S., Unzeta M. (2002). Neuroprotective Effect of the Monoamine Oxidase Inhibitor PF 9601N [N-(2-Propynyl)-2-(5-Benzyloxy-Indolyl) Methylamine] on Rat Nigral Neurons after 6-Hydroxydopamine-Striatal Lesion. Neurosci. Lett..

[95] Gardoni F., Morari M., Kulisevsky J., Brugnoli A., Novello S., Pisanò C.A., Caccia C., Mellone M., Melloni E., Padoani G. (2018). Safinamide Modulates Striatal Glutamatergic Signaling in a Rat Model of Levodopa-Induced Dyskinesia. J. Pharmacol. Exp. Ther..

[96] Sadeghian M., Mullali G., Pocock J.M., Piers T., Roach A., Smith K.J. (2016). Neuroprotection by Safinamide in the 6-Hydroxydopamine Model of Parkinson’s Disease. Neuropathol. Appl. Neurobiol..

[97] Sciaccaluga M., Mazzocchetti P., Bastioli G., Ghiglieri V., Cardinale A., Mosci P., Caccia C., Keywood C., Melloni E., Padoani G. (2020). Effects of Safinamide on the Glutamatergic Striatal Network in Experimental Parkinson’s Disease. Neuropharmacology.

[98] Huleatt P.B., Khoo M.L., Chua Y.Y., Tan T.W., Liew R.S., Balogh B., Deme R., Gölöncsér F., Magyar K., Sheela D.P. (2015). Novel Arylalkenylpropargylamines as Neuroprotective, Potent, and Selective Monoamine Oxidase B Inhibitors for the Treatment of Parkinson’s Disease. J. Med. Chem..

[99] Bar-Am O., Amit T., Kupershmidt L., Aluf Y., Mechlovich D., Kabha H., Danovitch L., Zurawski V.R., Youdim M.B.H., Weinreb O. (2015). Neuroprotective and Neurorestorative Activities of a Novel Iron Chelator-Brain Selective Monoamine Oxidase-A/Monoamine Oxidase-B Inhibitor in Animal Models of Parkinson’s Disease and Aging. Neurobiol. Aging.

[100] Bar Am O., Amit T., Youdim M.B.H. (2004). Contrasting Neuroprotective and Neurotoxic Actions of Respective Metabolites of Anti-Parkinson Drugs Rasagiline and Selegiline. Neurosci. Lett..

[101] Voronin M.V., Aksenova L.N., Buneena O.A., Medvedev A.E. (2009). Effect of Afobazole on Mitochondrial Monoamine Oxidase A Activity in Vitro. Bull. Exp. Biol. Med..

[102] Langston J.W., Ballard P., Tetrud J.W., Irwin I. (1983). Chronic Parkinsonism in Humans Due to a Product of Meperidine-Analog Synthesis. Science.

[103] Herraiz T., Guillén H. (2011). Inhibition of the Bioactivation of the Neurotoxin MPTP by Antioxidants, Redox Agents and Monoamine Oxidase Inhibitors. Food Chem. Toxicol..

[104] Langston J.W. (2017). The MPTP Story. J. Park. Dis..

[105] Meredith G.E., Rademacher D.J. (2011). MPTP Mouse Models of Parkinson’s Disease: An Update. J. Park. Dis..

[106] Mustapha M., Taib C.N.M. (2021). MPTP-Induced Mouse Model of Parkinson’s Disease: A Promising Direction for Therapeutic Strategies. Bosn. J. Basic. Med. Sci..

[107] Smeyne R.J., Jackson-Lewis V. (2005). The MPTP model of Parkinson’s disease. Mol. Brain Res..

[108] Kitayama S., Mitsuhata C., Davis S., Wang J.B., Sato T., Morita K., Uhl G.R., Dohi T. (1998). MPP+ Toxicity and Plasma Membrane Dopamine Transporter: Study Using Cell Lines Expressing the Wild-Type and Mutant Rat Dopamine Transporters. Biochim. Biophys. Acta.

[109] Schmidt N., Ferger B. (2001). Neurochemical Findings in the MPTP Model of Parkinson’s Disease. J. Neural Transm..

[110] Shanesazzade Z., Peymani M., Ghaedi K., Nasr Esfahani M.H. (2018). miR-34a/BCL-2 Signaling Axis Contributes to Apoptosis in MPP+ -Induced SH-SY5Y Cells. Mol. Genet. Genom. Med..

[111] Chun H.S., Gibson G.E., DeGiorgio L.A., Zhang H., Kidd V.J., Son J.H. (2001). Dopaminergic Cell Death Induced by MPP(+), Oxidant and Specific Neurotoxicants Shares the Common Molecular Mechanism. J. Neurochem..

[112] Van Raamsdonk J.M., Vega I.E., Brundin P. (2017). Oxidative Stress in Neurodegenerative Disease: Causation or Association?. Oncotarget.

[113] Niranjan R., Mishra K.P., Thakur A.K. (2018). Inhibition of Cyclooxygenase-2 (COX-2) Initiates Autophagy and Potentiates MPTP-Induced Autophagic Cell Death of Human Neuroblastoma Cells, SH-SY5Y: An Inside in the Pathology of Parkinson’s Disease. Mol. Neurobiol..

[114] Jiang P., Dickson D.W. (2018). Parkinson’s Disease: Experimental Models and Reality. Acta Neuropathol..

[115] McKinnon C., De Snoo M.L., Gondard E., Neudorfer C., Chau H., Ngana S.G., O’Hara D.M., Brotchie J.M., Koprich J.B., Lozano A.M. (2020). Early-Onset Impairment of the Ubiquitin-Proteasome System in Dopaminergic Neurons Caused by α-Synuclein. Acta Neuropathol. Commun..

[116] Kang S.S., Ahn E.H., Zhang Z., Liu X., Manfredsson F.P., Sandoval I.M., Dhakal S., Iuvone P.M., Cao X., Ye K. (2018). α-Synuclein Stimulation of Monoamine oxidase-B and Legumain Protease Mediates the Pathology of Parkinson’s Disease. EMBO J..

[117] Buneeva O.A., Kopylov A.T., Gnedenko O.V., Medvedeva M.V., Kapitsa I.G., Ivanova E.A., Ivanov A.S., Medvedev A.E. (2021). Changes in the mitochondrial subproteome of mouse brain Rpn13-binding proteins induced by the neurotoxin MPTP and the neuroprotector isatin. Biomed. Khim.

[118] Buneeva O.A., Medvedeva M.V., Kopylov A.T., Medvedev A.E. (2019). Ubiquitin Subproteome of Brain Mitochondria and Its Changes Induced by Experimental Parkinsonism and Action of Neuroprotectors. Biochem. Mosc..

[119] Buneeva O.A., Kopylov A.T., Nerobkova L.N., Kapitsa I.G., Zgoda V.G., Medvedev A.E. (2017). The effect of neurotoxin MPTP administration to mice on the proteomic profile of brain isatin-binding proteins. Biomed. Khim.

[120] Chin M.H., Qian W.-J., Wang H., Petyuk V.A., Bloom J.S., Sforza D.M., Laćan G., Liu D., Khan A.H., Cantor R.M. (2008). Mitochondrial Dysfunction, Oxidative Stress, and Apoptosis Revealed by Proteomic and Transcriptomic Analyses of the Striata in Two Mouse Models of Parkinson’s Disease. J. Proteome Res..

[121] Jin J., Meredith G.E., Chen L., Zhou Y., Xu J., Shie F.-S., Lockhart P., Zhang J. (2005). Quantitative Proteomic Analysis of Mitochondrial Proteins: Relevance to Lewy Body Formation and Parkinson’s Disease. Brain Res. Mol. Brain Res..

[122] Liu B., Shi Q., Ma S., Feng N., Li J., Wang L., Wang X. (2008). Striatal 19S Rpt6 Deficit Is Related to Alpha-Synuclein Accumulation in MPTP-Treated Mice. Biochem. Biophys. Res. Commun..

[123] Zhang X., Zhou J.-Y., Chin M.H., Schepmoes A.A., Petyuk V.A., Weitz K.K., Petritis B.O., Monroe M.E., Camp D.G., Wood S.A. (2010). Region-Specific Protein Abundance Changes in the Brain of MPTP-Induced Parkinson’s Disease Mouse Model. J. Proteome Res..

[124] Burté F., De Girolamo L.A., Hargreaves A.J., Billett E.E. (2011). Alterations in the Mitochondrial Proteome of Neuroblastoma Cells in Response to Complex 1 Inhibition. J. Proteome Res..

[125] Mingazov E.R., Khakimova G.R., Kozina E.A., Medvedev A.E., Buneeva O.A., Bazyan A.S., Ugrumov M.V. (2018). MPTP Mouse Model of Preclinical and Clinical Parkinson’s Disease as an Instrument for Translational Medicine. Mol. Neurobiol..

[126] Ugrumov M.V., Khaindrava V.G., Kozina E.A., Kucheryanu V.G., Bocharov E.V., Kryzhanovsky G.N., Kudrin V.S., Narkevich V.B., Klodt P.M., Rayevsky K.S. (2011). Modeling of Presymptomatic and Symptomatic Stages of Parkinsonism in Mice. Neuroscience.

[127] Langston J.W., Irwin I., Langston E.B., Forno L.S. (1984). Pargyline Prevents MPTP-Induced Parkinsonism in Primates. Science.

[128] Adeyemo O.M., Youdim M.B., Markey S.P., Markey C.J., Pollard H.B. (1993). L-Deprenyl Confers Specific Protection against MPTP-Induced Parkinson’s Disease-like Movement Disorder in the Goldfish. Eur. J. Pharmacol..

[129] Wu R.M., Murphy D.L., Chiueh C.C. (1996). Suppression of Hydroxyl Radical Formation and Protection of Nigral Neurons by L-Deprenyl (Selegiline). Ann. N. Y. Acad. Sci..

[130] Andoh T., Chock P.B., Murphy D.L., Chiueh C.C. (2005). Role of the Redox Protein Thioredoxin in Cytoprotective Mechanism Evoked by (-)-Deprenyl. Mol. Pharmacol..

[131] Heikkila R.E., Manzino L., Cabbat F.S., Duvoisin R.C. (1984). Protection against the Dopaminergic Neurotoxicity of 1-Methyl-4-Phenyl-1,2,5,6-Tetrahydropyridine by Monoamine Oxidase Inhibitors. Nature.

[132] Kupsch A., Sautter J., Götz M.E., Breithaupt W., Schwarz J., Youdim M.B., Riederer P., Gerlach M., Oertel W.H. (2001). Monoamine Oxidase-Inhibition and MPTP-Induced Neurotoxicity in the Non-Human Primate: Comparison of Rasagiline (TVP 1012) with Selegiline. J. Neural Transm..

[133] Baranyi M., Porceddu P.F., Gölöncsér F., Kulcsár S., Otrokocsi L., Kittel Á., Pinna A., Frau L., Huleatt P.B., Khoo M.-L. (2016). Novel (Hetero)Arylalkenyl Propargylamine Compounds Are Protective in Toxin-Induced Models of Parkinson’s Disease. Mol. Neurodegener..

[134] Youdim M.B., Gross A., Finberg J.P. (2001). Rasagiline [N-Propargyl-1R(+)-Aminoindan], a Selective and Potent Inhibitor of Mitochondrial Monoamine Oxidase B. Br. J. Pharmacol..

[135] Mandel S., Sagi Y., Amit T. (2007). Rasagiline Promotes Regeneration of Substantia Nigra Dopaminergic Neurons in Post-MPTP-Induced Parkinsonism via Activation of Tyrosine Kinase Receptor Signaling Pathway. Neurochem. Res..

[136] Sagi Y., Mandel S., Amit T., Youdim M. (2007). Activation of Tyrosine Kinase Receptor Signaling Pathway by Rasagiline Facilitates Neurorescue and Restoration of Nigrostriatal Dopamine Neurons in Post-MPTP-Induced Parkinsonism. Neurobiol. Dis..

[137] Gal S., Zheng H., Fridkin M., Youdim M.B.H. (2010). Restoration of Nigrostriatal Dopamine Neurons in Post-MPTP Treatment by the Novel Multifunctional Brain-Permeable Iron Chelator-Monoamine Oxidase Inhibitor Drug, M30. Neurotox. Res..

[138] Youdim M.B.H. (2012). M30, a Brain Permeable Multitarget Neurorestorative Drug in Post Nigrostriatal Dopamine Neuron Lesion of Parkinsonism Animal Models. Park. Relat. Disord..

[139] Liu Z., Cai W., Lang M., Yan R., Li Z., Zhang G., Yu P., Wang Y., Sun Y., Zhang Z. (2017). Neuroprotective Effects and Mechanisms of Action of Multifunctional Agents Targeting Free Radicals, Monoamine Oxidase B and Cholinesterase in Parkinson’s Disease Model. J. Mol. Neurosci..

[140] Muck-Seler D., Sagud M., Mustapic M., Nedic G., Babic A., Mihaljevic Peles A., Jakovljevic M., Pivac N. (2008). The Effect of Lamotrigine on Platelet Monoamine Oxidase Type B Activity in Patients with Bipolar Depression. Prog. Neuropsychopharmacol. Biol. Psychiatry.

[141] Lagrue E., Chalon S., Bodard S., Saliba E., Gressens P., Castelnau P. (2007). Lamotrigine Is Neuroprotective in the Energy Deficiency Model of MPTP Intoxicated Mice. Pediatr. Res..

[142] Lee C.S., Han E.S., Jang Y.Y., Han J.H., Ha H.W., Kim D.E. (2000). Protective Effect of Harmalol and Harmaline on MPTP Neurotoxicity in the Mouse and Dopamine-Induced Damage of Brain Mitochondria and PC12 Cells. J. Neurochem..

[143] Park T.H., Kwon O.S., Park S.Y., Han E.S., Lee C.S. (2003). N-Methylated Beta-Carbolines Protect PC12 Cells from Cytotoxic Effect of MPP+ by Attenuation of Mitochondrial Membrane Permeability Change. Neurosci. Res..

[144] Rajeswari A., Sabesan M. (2008). Inhibition of Monoamine Oxidase-B by the Polyphenolic Compound, Curcumin and Its Metabolite Tetrahydrocurcumin, in a Model of Parkinson’s Disease Induced by MPTP Neurodegeneration in Mice. Inflammopharmacology.

[145] Buneeva O., Kopylov A., Kapitsa I., Ivanova E., Medvedev A. (2020). Neuroprotective mechanisms of action of endogenous regulator isatin in MPTP induced Parkinsonism. Proceedings of the Neuroscience for Medicine and Psychology.

[146] Buneeva O., Kopylov A., Kapitsa I., Ivanova E., Zgoda V., Medvedev A. (2018). The Effect of Neurotoxin MPTP and Neuroprotector Isatin on the Profile of Ubiquitinated Brain Mitochondrial Proteins. Cells.

[147] Medvedev A.E., Buneeva O.A., Kopylov A.T., Tikhonova O.V., Medvedeva M.V., Nerobkova L.N., Kapitsa I.G., Zgoda V.G. (2017). Brain Mitochondrial Subproteome of Rpn10-Binding Proteins and Its Changes Induced by the Neurotoxin MPTP and the Neuroprotector Isatin. Biochemistry.

[148] Lee C.S., Han E.S., Lee W.B. (2003). Antioxidant Effect of Phenelzine on MPP+-Induced Cell Viability Loss in Differentiated PC12 Cells. Neurochem. Res..

[149] Bhattamisra S.K., Yap K.H., Rao V., Choudhury H. (2019). Multiple Biological Effects of an Iridoid Glucoside, Catalpol, and Its Underlying Molecular Mechanisms. Biomolecules.

[150] Xu G., Xiong Z., Yong Y., Wang Z., Ke Z., Xia Z., Hu Y. (2010). Catalpol Attenuates MPTP Induced Neuronal Degeneration of Nigral-Striatal Dopaminergic Pathway in Mice through Elevating Glial Cell Derived Neurotrophic Factor in Striatum. Neuroscience.

[151] Bi J., Wang X.-B., Chen L., Hao S., An L.-J., Jiang B., Guo L. (2008). Catalpol Protects Mesencephalic Neurons against MPTP Induced Neurotoxicity via Attenuation of Mitochondrial Dysfunction and MAO-B Activity. Toxicol. Vitr..

[152] Tian Y.-Y., Jiang B., An L.-J., Bao Y.-M. (2007). Neuroprotective Effect of Catalpol against MPP(+)-Induced Oxidative Stress in Mesencephalic Neurons. Eur. J. Pharmacol..

[153] Medvedev A., Kopylov A., Buneeva O., Kurbatov L., Tikhonova O., Ivanov A., Zgoda V. (2020). A Neuroprotective Dose of Isatin Causes Multilevel Changes Involving the Brain Proteome: Prospects for Further Research. Int. J. Mol. Sci..

[154] Buneeva O., Gnedenko O., Zgoda V., Kopylov A., Glover V., Ivanov A., Medvedev A., Archakov A. (2010). Isatin-Binding Proteins of Rat and Mouse Brain: Proteomic Identification and Optical Biosensor Validation. Proteomics.

[155] Medvedev A., Buneeva O., Gnedenko O., Ershov P., Ivanov A. (2018). Isatin, an Endogenous Nonpeptide Biofactor: A Review of Its Molecular Targets, Mechanisms of Actions, and Their Biomedical Implications. BioFactors.

[156] Yadav S., Dixit A., Agrawal S., Singh A., Srivastava G., Singh A.K., Srivastava P.K., Prakash O., Singh M.P. (2012). Rodent Models and Contemporary Molecular Techniques: Notable Feats yet Incomplete Explanations of Parkinson’s Disease Pathogenesis. Mol. Neurobiol..

[157] El-Gamal M., Salama M., Collins-Praino L.E., Baetu I., Fathalla A.M., Soliman A.M., Mohamed W., Moustafa A.A. (2021). Neurotoxin-Induced Rodent Models of Parkinson’s Disease: Benefits and Drawbacks. Neurotox. Res..

[158] Ibarra-Gutiérrez M.T., Serrano-García N., Orozco-Ibarra M. (2023). Rotenone-Induced Model of Parkinson’s Disease: Beyond Mitochondrial Complex I Inhibition. Mol. Neurobiol..

[159] Maegawa H., Niwa H. (2021). Generation of Mitochondrial Toxin Rodent Models of Parkinson’s Disease Using 6-OHDA, MPTP, and Rotenone. Methods Mol. Biol..

[160] Xiong N., Long X., Xiong J., Jia M., Chen C., Huang J., Ghoorah D., Kong X., Lin Z., Wang T. (2012). Mitochondrial Complex I Inhibitor Rotenone-Induced Toxicity and Its Potential Mechanisms in Parkinson’s Disease Models. Crit. Rev. Toxicol..

[161] Betarbet R., Canet-Aviles R.M., Sherer T.B., Mastroberardino P.G., McLendon C., Kim J.-H., Lund S., Na H.-M., Taylor G., Bence N.F. (2006). Intersecting Pathways to Neurodegeneration in Parkinson’s Disease: Effects of the Pesticide Rotenone on DJ-1, α-Synuclein, and the Ubiquitin–Proteasome System. Neurobiol. Dis..

[162] Greenamyre J.T., Betarbet R., Sherer T.B. (2003). The Rotenone Model of Parkinson’s Disease: Genes, Environment and Mitochondria. Park. Relat. Disord..

[163] Sherer T.B., Kim J.H., Betarbet R., Greenamyre J.T. (2003). Subcutaneous Rotenone Exposure Causes Highly Selective Dopaminergic Degeneration and Alpha-Synuclein Aggregation. Exp. Neurol..

[164] Subhan I., Siddique Y.H. (2024). Effect of Rotenone on the Neurodegeneration among Different Models. Curr. Drug Targets.

[165] Elmorsy E., Al-Ghafari A., Al Doghaither H., Hashish S., Salama M., Mudyanselage A.W., James L., Carter W.G. (2023). Differential Effects of Paraquat, Rotenone, and MPTP on Cellular Bioenergetics of Undifferentiated and Differentiated Human Neuroblastoma Cells. Brain Sci..

[166] Radad K., Al-Shraim M., Al-Emam A., Wang F., Kranner B., Rausch W.-D., Moldzio R. (2019). Rotenone: From Modelling to Implication in Parkinson’s Disease. Folia Neuropathol..

[167] Sanders L.H., Timothy Greenamyre J. (2013). Oxidative Damage to Macromolecules in Human Parkinson Disease and the Rotenone Model. Free Radic. Biol. Med..

[168] Buneeva O.A., Kapitsa I.G., Kazieva L.S., Vavilov N.E., Zgoda V.G. (2023). Quantitative Changes of Brain Isatin-Binding Proteins of Rats with the Rotenone-Induced Experimental Parkinsonism. Biomed. Khim.

[169] Gielisch I., Meierhofer D. (2015). Metabolome and Proteome Profiling of Complex I Deficiency Induced by Rotenone. J. Proteome Res..

[170] Jin J., Davis J., Zhu D., Kashima D.T., Leroueil M., Pan C., Montine K.S., Zhang J. (2007). Identification of Novel Proteins Affected by Rotenone in Mitochondria of Dopaminergic Cells. BMC Neurosci..

[171] Zhou Y., Gu G., Goodlett D.R., Zhang T., Pan C., Montine T.J., Montine K.S., Aebersold R.H., Zhang J. (2004). Analysis of α-Synuclein-Associated Proteins by Quantitative Proteomics*[Boxs]. J. Biol. Chem..

[172] Buneeva O.A., Kapitsa I.G., Kazieva L.S., Vavilov N.E., Zgoda V.G., Medvedev A.E. (2024). The Delayed Effect of Rotenone on the Relative Content of Brain Isatin-Binding Proteins of Rats with Experimental Parkinsonism. Biomed. Khim.

[173] Shimizu K., Ohtaki K., Matsubara K., Aoyama K., Uezono T., Saito O., Suno M., Ogawa K., Hayase N., Kimura K. (2001). Carrier-Mediated Processes in Blood–Brain Barrier Penetration and Neural Uptake of Paraquat. Brain Res..

[174] Kumar A., Ganini D., Mason R.P. (2016). Role of Cytochrome c in α-Synuclein Radical Formation: Implications of α-Synuclein in Neuronal Death in Maneb- and Paraquat-Induced Model of Parkinson’s Disease. Mol. Neurodegener..

[175] Jagmag S.A., Tripathi N., Shukla S.D., Maiti S., Khurana S. (2015). Evaluation of Models of Parkinson’s Disease. Front. Neurosci..

[176] See W.Z.C., Naidu R., Tang K.S. (2022). Cellular and Molecular Events Leading to Paraquat-Induced Apoptosis: Mechanistic Insights into Parkinson’s Disease Pathophysiology. Mol. Neurobiol..

[177] Sharma P., Mittal P. (2024). Paraquat (Herbicide) as a Cause of Parkinson’s Disease. Park. Relat. Disord..

[178] Zhang X., Thompson M., Xu Y. (2016). Multifactorial Theory Applied to the Neurotoxicity of Paraquat and Paraquat-Induced Mechanisms of Developing Parkinson’s Disease. Lab. Investig..

[179] Dixit A., Srivastava G., Verma D., Mishra M., Singh P.K., Prakash O., Singh M.P. (2013). Minocycline, Levodopa and MnTMPyP Induced Changes in the Mitochondrial Proteome Profile of MPTP and Maneb and Paraquat Mice Models of Parkinson’s Disease. Biochim. Biophys. Acta (BBA)—Mol. Basis Dis..

[180] Patel S., Sinha A., Singh M.P. (2007). Identification of Differentially Expressed Proteins in Striatum of Maneb-and Paraquat-Induced Parkinson’s Disease Phenotype in Mouse. Neurotoxicol Teratol..

[181] Saravanan K.S., Sindhu K.M., Senthilkumar K.S., Mohanakumar K.P. (2006). L-Deprenyl Protects against Rotenone-Induced, Oxidative Stress-Mediated Dopaminergic Neurodegeneration in Rats. Neurochem. Int..

[182] Liu B., Lv C., Zhang J., Liu Y., Sun J., Cheng X., Mao W., Ma Y., Li S. (2017). Effects of Eldepryl on Glial Cell Proliferation and Activation in the Substantia Nigra and Striatum in a Rat Model of Parkinson’s Disease. Neurol. Res..

[183] Liu B., Sun J., Zhang J., Mao W., Ma Y., Li S., Cheng X., Lv C. (2015). Autophagy-Related Protein Expression in the Substantia Nigra and Eldepryl Intervention in Rat Models of Parkinson’s Disease. Brain Res..

[184] Liou H.-H., Chen R.-C., Chen T.H.-H., Tsai Y.-F., Tsai M.-C. (2001). Attenuation of Paraquat-Induced Dopaminergic Toxicity on the Substantia Nigra by (−)-Deprenyl in Vivo. Toxicol. Appl. Pharmacol..

[185] Chau K.Y., Cooper J.M., Schapira A.H.V. (2010). Rasagiline Protects against Alpha-Synuclein Induced Sensitivity to Oxidative Stress in Dopaminergic Cells. Neurochem. Int..

[186] Buneeva O.A., Kapitsa I.G., Zgoda V.G., Medvedev A.E. (2023). Neuroprotective Effects of Isatin and Afobazole in Rats with Rotenone-Induced Parkinsonism Are Accompanied by Increased Brain Levels of Triton X-100 Soluble Alpha-Synuclein. Biomed. Khim.

[187] Buneeva O.A., Kapitsa I.G., Kazieva L.S., Vavilov N.E., Zgoda V.G., Medvedev A.E. (2024). The Neuroprotective Effect of Isatin in the Rotenone-Induced Model of Parkinonism in Rats: The Study of Delayed Effects. Biomed. Khim..

[188] Fitzgerald J.C., Ugun-Klusek A., Allen G., De Girolamo L.A., Hargreaves I., Ufer C., Abramov A.Y., Billett E.E. (2014). Monoamine Oxidase-A Knockdown in Human Neuroblastoma Cells Reveals Protection against Mitochondrial Toxins. FASEB J..

[189] Anderzhanova E.A., Bächli H., Buneeva O.A., Narkevich V.B., Medvedev A.E., Thoeringer C.K., Wotjak C.T., Kudrin V.S. (2013). Strain Differences in Profiles of Dopaminergic Neurotransmission in the Prefrontal Cortex of the BALB/C vs. C57Bl/6 Mice: Consequences of Stress and Afobazole. Eur. J. Pharmacol..

[190] Naoi M., Maruyama W., Shamoto-Nagai M. (2020). Rasagiline and Selegiline Modulate Mitochondrial Homeostasis, Intervene Apoptosis System and Mitigate α-Synuclein Cytotoxicity in Disease-Modifying Therapy for Parkinson’s Disease. J. Neural Transm..

[191] Panova N.G., Axenova L.N., Medvedev A.E. (2000). The Stimulating Effects of Ethanol Consumption on Synthesis of Rat Brain Monoamine Oxidases and Their Sensitivity to the Irreversible Inhibitor, Pargyline. Neurosci. Lett..

[192] Crumeyrolle-Arias M., Medvedev A., Cardona A., Barritault D., Glover V. (2003). In Situ Imaging of Specific Binding of [3H]Isatin in Rat Brain. J. Neurochem..

[193] Kunikowska G., Gallagher I., Glover V., Clow A., Jenner P. (2002). Effects of Short- and Long-Term (−)-Deprenyl Administration on mRNA for Copper, Zinc- and Manganese-Superoxide Dismutase and Glutathione Peroxidase in Rat Brain. Brain Res..

[194] Fedchenko V., Globa A., Kaloshin A., Kapitsa I., Nerobkova L., Val’dman E., Buneeva O., Glover V., Medvedev A. (2008). The Effect of Short-Term Administration of (-)-Deprenyl and Isatin on the Expressions of Some Genes in the Mouse Brain Cortex. Med. Sci. Monit..

[195] Itzhak Y., Kassim C.O. (1990). Clorgyline Displays High Affinity for Sigma Binding Sites in C57BL/6 Mouse Brain. Eur. J. Pharmacol..

[196] Akao Y., Maruyama W., Yi H., Shamoto-Nagai M., Youdim M.B.H., Naoi M. (2002). An Anti-Parkinson’s Disease Drug, N-Propargyl-1(R)-Aminoindan (Rasagiline), Enhances Expression of Anti-Apoptotic Bcl-2 in Human Dopaminergic SH-SY5Y Cells. Neurosci. Lett..

[197] Bar-Am O., Weinreb O., Amit T., Youdim M.B.H. (2005). Regulation of Bcl-2 Family Proteins, Neurotrophic Factors, and APP Processing in the Neurorescue Activity of Propargylamine. FASEB J..

[198] Weinreb O., Bar-Am O., Amit T., Chillag-Talmor O., Youdim M.B.H. (2004). Neuroprotection via Pro-Survival Protein Kinase C Isoforms Associated with Bcl-2 Family Members. FASEB J..

[199] Naoi M., Maruyama W., Shamoto-Nagai M. (2018). Type A and B Monoamine Oxidases Distinctly Modulate Signal Transduction Pathway and Gene Expression to Regulate Brain Function and Survival of Neurons. J. Neural Transm..

[200] Moura D.J., Richter M.F., Boeira J.M., Pêgas Henriques J.A., Saffi J. (2007). Antioxidant Properties of Beta-Carboline Alkaloids Are Related to Their Antimutagenic and Antigenotoxic Activities. Mutagenesis.

[201] Buneeva O., Medvedev A. (2022). Atypical Ubiquitination and Parkinson’s Disease. Int. J. Mol. Sci..

[202] Buneeva O.A., Medvedev A.E. (2016). Atypical Ubiquitination of Proteins. Biomeditsinskaia Khimiia.

[203] Buneeva O.A., Kopylov A.T., Tikhonova O.V., Zgoda V.G., Medvedev A.E., Archakov A.I. (2012). Effect of Affinity Sorbent on Proteomic Profiling of Isatin-Binding Proteins of Mouse Brain. Biochemistry.

[204] Dutta G., Zhang P., Liu B. (2008). The Lipopolysaccharide Parkinson’s Disease Animal Model: Mechanistic Studies and Drug Discovery. Fundam. Clin. Pharmacol..

[205] Kang S., Noh Y., Oh S.J., Yoon H.J., Im S., Kwon H.T., Pak Y.K. (2023). Neuroprotective Effects of Aldehyde-Reducing Composition in an LPS-Induced Neuroinflammation Model of Parkinson’s Disease. Molecules.

[206] Ostadkarampour M., Putnins E.E. (2021). Monoamine Oxidase Inhibitors: A Review of Their Anti-Inflammatory Therapeutic Potential and Mechanisms of Action. Front. Pharmacol..

[207] Gelders G., Baekelandt V., Van der Perren A. (2018). Linking Neuroinflammation and Neurodegeneration in Parkinson’s Disease. J. Immunol. Res..

